# Hierarchical Sequencing and Feedforward and Feedback Control Mechanisms in Speech Production: A Preliminary Approach for Modeling Normal and Disordered Speech

**DOI:** 10.3389/fncom.2020.573554

**Published:** 2020-11-11

**Authors:** Bernd J. Kröger, Catharina Marie Stille, Peter Blouw, Trevor Bekolay, Terrence C. Stewart

**Affiliations:** ^1^Department for Phoniatrics, Pedaudiology and Communication Disorders, Medical Faculty, RWTH Aachen University, Aachen, Germany; ^2^Applied Brain Research, Waterloo, ON, Canada; ^3^Centre for Theoretical Neuroscience, University of Waterloo, Waterloo, ON, Canada; ^4^National Research Council of Canada, University of Waterloo Collaboration Centre, Waterloo, ON, Canada

**Keywords:** neurocomputational model, computer simulation, speech processing, speech disorders, aphasia, hierarchical sequencing, neural engineering framework (NEF), semantic pointer architecture (SPA)

## Abstract

Our understanding of the neurofunctional mechanisms of speech production and their pathologies is still incomplete. In this paper, a comprehensive model of speech production based on the Neural Engineering Framework (NEF) is presented. This model is able to activate sensorimotor plans based on cognitive-functional processes (i.e., generation of the intention of an utterance, selection of words and syntactic frames, generation of the phonological form and motor plan; feedforward mechanism). Since the generation of different states of the utterance are tied to different levels in the speech production hierarchy, it is shown that different forms of speech errors as well as speech disorders can arise at different levels in the production hierarchy or are linked to different levels and different modules in the speech production model. In addition, the influence of the inner feedback mechanisms on normal as well as on disordered speech is examined in terms of the model. The model uses a small number of core concepts provided by the NEF, and we show that these are sufficient to create this neurobiologically detailed model of the complex process of speech production in a manner that is, we believe, clear, efficient, and understandable.

## Introduction

This paper provides an overview of the neurolinguistic part of speech production, from initiation through formulation to implementation of the cognitive specification of an utterance in a phonological form (Levelt, 1989; Levelt et al., 1999; Indefrey and Levelt, 2004). First, the intention, the message, or the amount of information that the speaker wants to pass on to a communication partner is activated (i.e., a preverbal message). This information corresponds to a selection of semantic concepts (e.g., “boy, dog, hunting”) and a concept frame (e.g., “action, action executor, action object”). As part of the formulation, the language-specific knowledge repository (i.e., the mental lexicon) is then accessed and a word or lemma is assigned to each concept. In addition, every lemma is integrated into a grammatical-syntactic framework (e.g., “subject—predicate—object,” here: “boy—hunt—dog”) such that the selected words are inflected, functional words are added, and the words are ordered as a sentence in a defined sequence (here: “the boy is chasing the dog”). The formulation includes phonological encoding (i.e., access to the phonological form of each word), along with re-syllabification of the entire utterance, which may become necessary due to the inflection and sequencing of the words (e.g., in English: /he/passed/us/ -> /hi/pEs/t@s/; phonological transcriptions are embraced by /…/ and written in SAMPA-notation here (SAMPA, 2005).

The purely cognitive-symbolic representation of an utterance must be transformed into a movement sequence for the lips, tongue, soft palate, vocal folds, and chest (or respiratory system) as part of the articulation. For this purpose, the existence of a mental syllable memory (“mental syllabary”) is assumed (Cholin, 2008), which converts the phonological representation into motor commands, which then lead to articulation movements and are converted by the articulation apparatus into an acoustic speech signal. While the mental lexicon is a knowledge repository for cognitive entities, the mental syllabary can be viewed as a knowledge and skill repository for the motor realization of syllables based on phonological input entities. All common syllables of the speaker's practiced language are coded here in the form of motor plans. For each of these motor plans for a syllable there is at least one auditory and one somatosensory target representation with which the correct articulation of the syllable can then be checked (Guenther et al., 2006; Kröger et al., 2010, 2019; Guenther and Vladusich, 2012). Extending these models by implementing the concept of speech gestures (Goldstein and Fowler, 2003; Goldstein et al., 2006), a motor plan can be understood as a set of gestures in which the timing, i.e., the temporal coordination of all syllable speech movement units (“speech gestures,” e.g., consonant closure gestures, vocal opening gestures) is fixed. Spelling out the entire repertoire of gestures for a target language (e.g., for German) is given by Kröger and Birkholz (2007) and by Kröger and Bekolay (2019, p. 20).

The speech production model defined above includes six levels: a concept level, a lemma level, a level of phonological forms, a motor plan level, an articulatory level, and an acoustic signal level. Furthermore, in addition to the top-down processing within this model, several feedback mechanisms are included based on the information or data generated during top-down or “feed-forward directed” production. This information is passed on bottom-up in a “feedback” manner from lower to higher levels (Postma, 2000). A well-known and important feedback mechanism is that of controlling the self-generated acoustic speech signal by self-perception. If this signal does not meet the expectations of the speaker, a new and corrected realization of the utterance can be produced. The motor, phonological or conceptual realization of the utterance, which is feed bottom-up on the basis of the acoustic-auditory signal, can be compared with the previously planned motor, phonological, or conceptual form of the utterance and checked for differences in order to identify possible production errors at certain levels within the speech production process.

Hickok (2012) assumes that auditory feedback at the motor level is more used to correct syllabic motor plans, while somatosensory feedback, i.e., the feedback of tactile and proprioceptive information, serves to control the sound-related speech gestures (“speech movement units,” Kröger and Bekolay, 2019, p. 17ff). While auditory feedback is too slow to effect real-time corrections, somatosensory feedback control of speech gestures can lead to real-time corrections. Since speaking can also take place internally (inner speech, Postma, 2000), the control of the phonological as well as of the conceptual realization of an utterance can already be achieved if the inner realization of the utterance at the phonological level is realized and if perhaps in addition motor and sensory correlates of the syllables of the utterance are activated as well, but without concrete articulatory execution. At this point in time an internal feedback process can be initiated (Postma, 2000), leading to a bottom-up feedback process involving the produced forms at the phonological, lemma, and concept levels of the production hierarchy. This feedback mechanism is fast and can lead to real-time corrections at the cognitive level in the production hierarchy.

A quantitative formulation of the modules and levels of language production described above is achieved in particular by two models. The model WEAVER (Word Encoding by Activation and VERification; Roelofs, 1992, 1997, 2014) is a quantitative formulation and computer implementation of the cognitive modules. This model is a quantitative implementation of the approach described by Levelt et al. (1999), which comprises all levels from conceptualization to the realization of the phonological form of an utterance. The model is able to simulate behavioral data for word production, for example by correctly simulating picture naming tasks, word comprehension tasks, and nonsense-word (“logatome”) repetition tasks. This applies both to behavioral data from healthy people as well as behavioral speech data gathered from patients suffering from aphasia. In particular, six different forms of aphasia, namely Broca-, Wernicke-, transcortical-motor, transcortical-sensory, mixed and conduction aphasias can be modeled using this model (Roelofs, 2014).

The DIVA model (Directions Into Velocities of Articulators: Guenther, 2006; Guenther et al., 2006; Golfinopoulos et al., 2010; Guenther and Vladusich, 2012) also describes a concrete computer-implemented model of speech production. This model starts with the phonological level and implements all phonetic and motor processes involved in speech production down to articulation, generation of the acoustic signal, and processing of somatosensory and auditory feedback information for error correction on the speech signal just realized. The model is able to correctly simulate syllables, words, and short multi-syllable utterances. In addition, by means of the modeling of the somatosensory and auditory feedback, this model is able to implement the generation of typical modifications to the already learned and automated motor commands (“forward control”) that are necessary in the case articulatory or sensory disturbances leading to differences between the signals that have just been generated and those that are expected. This “re-learning” of articulation in cases of unexpected but longer lasting disturbances of the articulatory and sensory conditions (e.g., “bite-block” cases or “formant shifting”) is in accordance with behavioral experimental data. In more recent simulation studies, the DIVA model has also been successfully used to simulate the articulation of patients suffering from apraxia of speech from different forms of dysarthrias (Kearney and Guenther, 2019; Miller and Guenther, 2020).

The above-mentioned model approaches are already capable of imitating behavioral experimental data (Guenther and Vladusich, 2012; Roelofs, 2014). In addition, the individual modules mentioned in these models can be assigned to defined cortical and subcortical regions (Indefrey and Levelt, 2004; Golfinopoulos et al., 2010).

The models mentioned above are, however, quite simple connectionist models (for a differentiation of simple connectionist models and spiking neuron models see: Kröger and Bekolay, p. 133 ff). It is assumed that each concept, each lemma, and each phonological form is described by a defined “node” on the corresponding level of a neural network representing the production model. These nodes can be “activated” and the activation of individual nodes can be spread to other nodes by means of “edges.” In contrast the modeling approach for speech production described in this publication is based on the NEF-SPA (Neural Engineering Framework, see Eliasmith and Anderson, 2004; and Semantic Pointer Architecture, see: Eliasmith et al., 2012; Eliasmith, 2013; Stewart and Eliasmith, 2014). Here, neural states are represented by biologically inspired neural activity patterns occurring in defined neural state buffers. These neural state buffers consist of several thousand specifically implemented “leaky-integrate-and-fire” neurons (Eliasmith, 2013). Each state buffer is capable of representing different neural states, and each state can be identified by its own characteristic “neural activity pattern” occurring in that neural state buffer. Different neural activation patterns represent different cognitive, motor, or sensory states corresponding to concepts, lemmas, and phonological forms, as well as motor, auditory, and sensory states of syllables. Thus, each state is not realized by a node (by a “local representation” of states) in terms of our NEF-SPA approach, but by an activation pattern of many neurons associated with each other within a specific state buffer (i.e., by a “distributed representation” of states).

**Figure 1:**
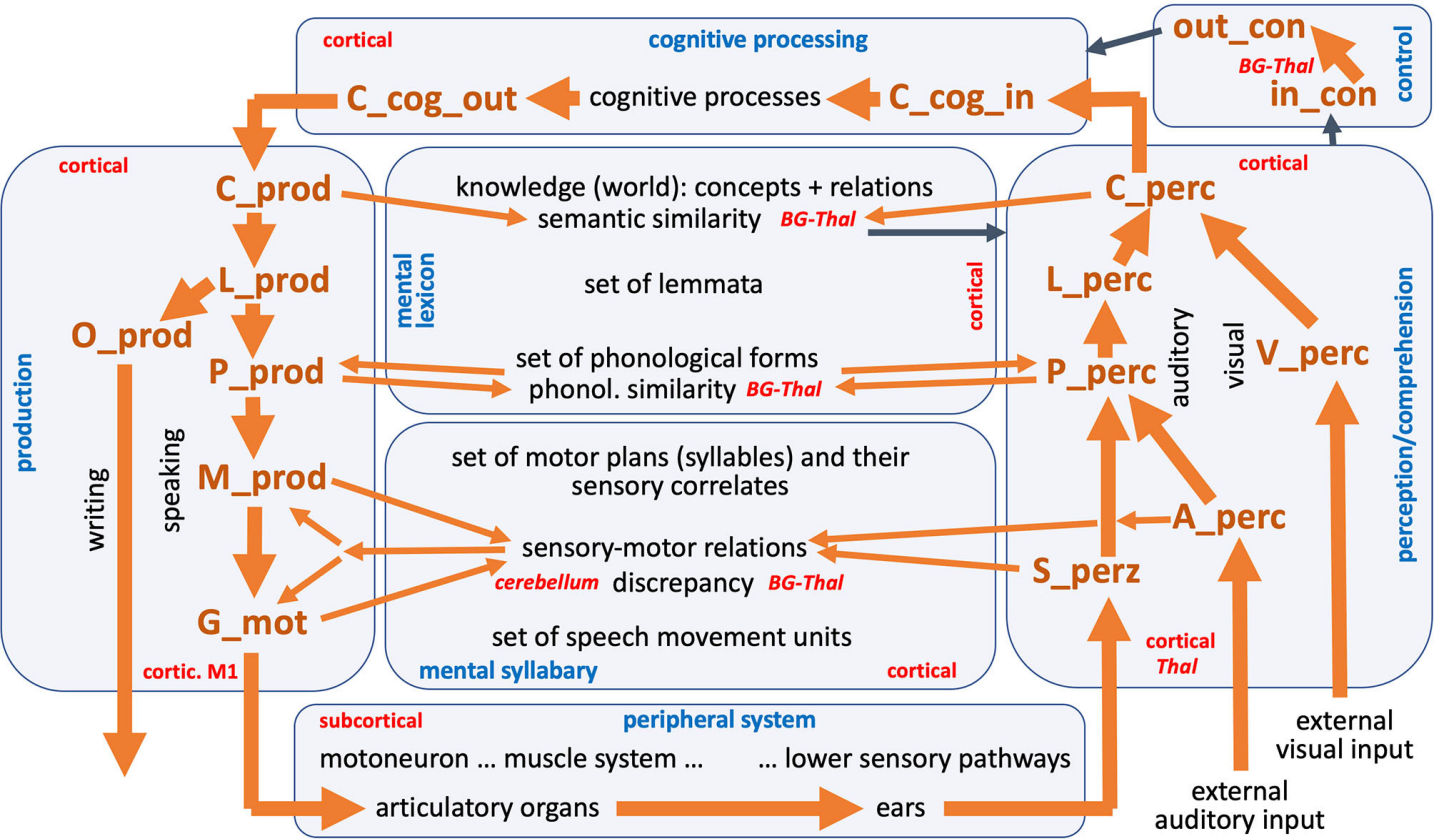
The entire neural model of speech processing. Blue, names of modules; red, hypothetical anatomical location of modules or parts of modules; black, production and perception modalities within production and perception pathway; black, different state levels within the knowledge and skill repository (mental lexicon and mental syllabary); black, peripheral sub-systems and cognitive processes; back, external visual and auditory input; brown, names of state buffers (for description of all state buffers see text and see Table A1 in Appendix). Thick brown arrows indicates the main processing or feedback loop (see text); thin brown arrows indicate all other feedback loops; thin black arrows indicate the interaction between control module with all other modules.

In addition, as already mentioned above, the model neurons within the NEF-SPA approach are implemented here in a biologically inspired manner. Furthermore, the modeling of neuronal connections using associative memories and the modeling of “binding” or “unbinding” of states (i.e., modeling of short-term relationships between states, e.g., for two concepts such as “blue” and “ball” to “blue ball”) by using special processing buffers (binding and unbinding buffers) is also biologically motivated (Stewart and Eliasmith, 2014). Thus, neural connections exist not necessarily between all neurons of two buffers, but between all neurons of one buffer (input buffer, holding the neural activity pattern of the input state) and all input neurons of a processing buffer as well as between all output neurons of that processing buffer and all neurons of the second buffer (output buffer, holding the neural activation pattern of the transformed or processed state). This modeling of transformations of states by interconnected processing buffers between state buffers is indicted by arrows in Figure 1, e.g., for the transformations of neural states from concept buffers to lemma buffers, or from lemma buffers to phonological form buffers on the production as well as on the perception side. This holds as well for those neural connections which implement complex transformations of states from one level to another level within the speech production model as well as for clean-up processes (Stewart and Eliasmith, 2014). The NEF-SPA approach is explained more fully below in the context of our speech production model. The NEF-SPA approach also allows a clearly structured implementation of neurobiological processes for modeling different behavioral scenarios because it includes a biologically motivated account of the selection of actions and thus of the control of neural processes (Stewart and Eliasmith, 2014). Thus, the NEF-SPA approach comprises a cortico-cortical circuit including basal ganglia and thalamus, biologically realistic neural firing processes for individual neurons, and biologically inspired modeling of the dynamics of information transfer at different types of synapses (Eliasmith, 2013; Stewart and Eliasmith, 2014). Such biologically inspired modeling of neural processes directly defines the timing of all neural processes in an unconstrained way.

It is the aim of this publication to describe a new comprehensive model of speech production based on the principles of the NEF-SPA. As explained below, this model is able to replicate behavioral data convincingly, by simulating both non-disordered as well as disordered speech realistically. Parts of the model have already been implemented and used for simulation experiments in earlier studies (Kröger et al., 2016a,b; Stille et al., 2019) but for the first time a comprehensive overview of the complete model is given in this publication in the next chapter.

## Method: Description of the Computer-Implemented Model

### The Entire Model: Feed-Forward-, Feedback-, and Learning-Processes

The model presented here comprises seven modules, namely the production channel, the perception channel, a knowledge repository and a skill repository that includes world knowledge and vocabulary knowledge within the mental lexicon and speaking skills in the mental syllabary, a module for the cognitive processing of input information and for the preparation of output information, a module for controlling all neural processes, and a module for the implementation of primary motor control commands and their muscle activation patterns into speech articulation with subsequent generation of sensory feedback signals for processing in the perception channel, called peripheral system (Figure 1).

The current version of our speech processing model is able to produce single words. The process of word initiation (Levelt et al., 1999) corresponds to the activation of a concept in the cognitive state memory C_cog_out (“C” means “concept;” the abbreviations and names for all state buffers are listed in Table A1 in the Appendix). In the NEF-SPA approach, all neural state buffers are able to activate S-pointers. An S-pointer represents a cognitive, motor, or sensory state (e.g., a word, a motor plan, or an auditory realization of a word or syllable). In the NEF-SPA approach, a number of S-pointers can be understood mathematically as D-dimensional vectors and, on the other hand, as activity patterns representing neuronal states in state buffers consisting of D ^*^ N leaky-integrate-and-fire neurons (D = 64 and N = 100 in the case of speech for processing a small mental lexicon; see Stille et al., 2019). Each individual dimension of an S-pointer vector (i.e., each numeric value) is encoded as the activation state of a neuron ensemble consisting of N neurons. D neuron ensembles build up a complete neural state buffer. A defined neural activity pattern in a neural state buffer consisting of D ^*^ N neurons can thus be assigned to each cognitive, motor, or sensory state via the mathematical construct of the vector representation (Kröger and Bekolay, 2019, p. 175ff).

An S-pointer network (Kröger and Bekolay, p. 206ff) also enables the inclusion of relations between S-pointers. At the concept level, this means that relations like “is a” put two S-pointers like “boy” and “human” in closer relation to each other than for example “boy” and “animal.” In our model, the development of such relations occurs at the level of concepts (world knowledge: concepts and their relations, Figure 1). In addition, relations can also be established at the level of phonological forms. Relationships are used here to relate similar phonological forms, such as all phonological forms for syllables that begin with /sk/ (e.g., /sku/ for the first syllable of the word “squirrel” and for example /skEIt/ for the word “skate”).

States are represented on the mathematical level by S-pointers of length one (Eliasmith, 2013; Stewart and Eliasmith, 2014; Kröger and Bekolay, 2019, p. 175ff). The definition of a set of S-pointers thus corresponds to the definition of a set of unit vectors in a D-dimensional vector space. In the case of independent S-pointers (independent states), these vectors are spaced as far apart as possible. In this way, a constant distance between the end points of the state vectors on the unit sphere of the D-dimensional vector space is realized, which on the mathematical level corresponds to a minimization of the DOT product between S-pointers (i.e., the cosine product between two vectors; Stewart and Eliasmith, 2014) for all combinations of S-pointers occurring at one specific level of the model (concepts, lemmata, phonological forms etc.). In the case of the definition of S-pointer networks, however, the relations between S-pointers are taken into account. Here, related S-pointers point in similar directions in the D-dimensional vector space and thus combinations of S-pointers that are in relation with each other have a higher DOT product and thus have a smaller distance to each other in comparison to other S-Pointer combinations on a specific model level (Kröger and Bekolay, 2019, p. 206ff). In terms of neuronal states, this means that activations of an S-pointer from an S-pointer network always co-activate those S-pointers or those corresponding states that are related to this S-pointer. Thus, a semantic S-Pointer network is able, for example, to represent our world knowledge, because this knowledge can be seen as a set of S-pointers and S-pointer relations.

S-pointers are of unit length (length one) in most cases, indicating a “normal activation strength of the appropriate neural state” or “clear activation” of that neural state. But after some processing steps the activity of neural states can decrease (from the mathematical viewpoint: its S-pointer decreases in length). This can be overcome by adding clean-up processes (Stewart and Eliasmith, 2014) in order to always clearly identify a cognitive, sensory, or motor state during neural processing.

The transformations of neural states between individual state buffers in the production channel [concept level (C_prod) -> lemma level (L_prod) -> phonological level (P_prod) -> motor plan level (M_prod)] as well as in the perception channel [primary sensory level of the somatosensory, auditory or visual signal (S_perc, A_perc and V_perc) -> phonological level (P_perc) -> lemma level (L_perc) -> concept level (C_perc)] as well as from the perception channel to the module of cognitive processing (C_perc -> C_cog_in) and from the module of cognitive processing to the production channel (C_cog_out -> C_prod) is realized in the NEF-SPA approach by using associative memories (Stewart and Eliasmith, 2014; Kröger and Bekolay, 2019, p. 186ff; for the definition of the neural buffers and their abbreviations see Table A1, Appendix). These associative memories are part of a long-term memory developed by learning. The memories contain the information about which concept has to be transformed into which lemma, which lemma into which phonological form etc. In the production channel, an activated concept is thus passed on from the state buffer C_cog_out to the state buffer C_prod, then by means of a neural transformation network including associative memories into the activation pattern of the associated lemma and then further transferred into the associated phonological form. The activations occur in state buffers L_prod for the lemma followed by the state memory P_prod for the phonological form (see Figure 1). If there is a one-syllable word, the associated motor plan is activated in the state memory M_prod in the same way using an associative memory. In the case of multi-syllable words, however, a temporal coordination of the sequence of syllables must also be implemented, which is done via the control module (see Kröger and Bekolay, 2019, p. 203ff).

To locate the state buffers as well as the associative memories in the brain it can be assumed: (i) all neuronal state buffers and associative memories of the auditory perception channel are located in the temporal lobe; (ii) all state buffers and associative memories of the visual perception channel are located in the occipital lobe toward the temporal lobe; and (iii) all neuronal state buffers and associative memories of the production channel are located in the frontal lobe, with strong neuronal association fibers running from the Broca area of the frontal lobe to the Wernicke area of the temporal lobe. Thus, motor information for syllables (motor plans) stored in the mental syllabary (in the Broca area of the frontal lobe) and their sensory correlates can be located not only in the frontal lobe but also in the temporal lobe (area Spt, see Hickok, 2012).

For each syllable, an associated motor plan is activated from the mental syllabary in the neuronal state buffer M_prod (Figure 1). All syllables which are represented in the mental syllabary are already practiced by the speaker during the course of language acquisition, such that it can be assumed that in addition to the motor representations, there is also an auditory (“A_perz”) and a somatosensory representation (“S_perz”) stored in the mental syllabary for each already known and already trained syllable (see perception channel in Figure 1). The activated motor plans can therefore also be referred to as sensorimotor representations for each syllable. On the side of the production channel, these plans specify the temporal coordination of all speech movement units occurring within the syllable (Figure 1: “G_mot,” with G for “gesture”). Speech movement units (gestures) are subordinate motor units (simple, purposeful articulator movements) that can be directly assigned to a goal-directed speech movement such as a labial closing gesture for realizing a /b/, /m/ or /p/, a glottal opening gesture for realizing an unvoiced sound (e.g., /p/, /f/, /s/, /t/ or /k/), or a velopharyngeal opening gesture for realizing a nasal sound (e.g., /n/ or /m/; see Kröger and Bekolay, 2019, p. 17ff).

In addition to the production channel, the perception channel is of crucial importance for speech production, since sensory feedback signals are processed in this channel. This results in a “large processing loop” that can be found in the model (Figure 1). This large processing loop can be explained by taking into consideration two process scenarios that often occur in speech production. The first process scenario is “direct word production,” which begins with the self-induced generation of an utterance; i.e., one word in case of our current model. Here, an intention realized as a semantic concept (C_cog_out) is forwarded to the concept level of the mental lexicon in the production channel (C_prod) and from there is forwarded through the production channel without intervention by the control module of the model (Figure 1). After running through an associative memory it leads to the activation of a lemma (L_prod), then, after passing through another associative memory, to the activation of a phonological form (P_prod), to the activation of a sequence of motor plans (M_prod) for each syllable in the word, and to the activation of a set of temporally coordinated speech movement units (SMUs or gestures, G_mot, see Kröger and Bekolay, 2019, p. 17ff). The resulting articulatory movement sequences leads to a generation if somatosensory and auditory feedback information activated in S_perc and A_perc, which then can be processed within the perception channel and be compared with the sensory expectations for the production of the word via the mental syllabary and mental lexicon.

A second process scenario that occurs frequently, a word repetition task, begins within the perception channel as part of the “large processing loop.” Based on an externally generated visual or auditory stimulus (V_perc, A_perc), a concept (C_perc) is activated, which can then be passed on directly to the production channel without further cognitive processing. In the further course of this paper, we differentiate between a word repetition task as was just mentioned here, and a logatome repetition task (logatomes = meaningless words or syllables), that involves repeating syllables or nonsense words without activating the mental lexicon, since nonsense words are not represented in the mental lexicon. In this second case, the auditory input signal, after recognizing its phonological structure (activation at level P_perc), is passed on directly to the phonological level of the production channel (P_prod) (shortcut from perception to production side on the phonological level; see thin brown arrows on that level in Figure 1).

Moreover, it should be noted that in a more complete model, in addition to the visual part of the perception channel (V_perc -> C_perc), there would also be an orthographic part for written language, or for the conversion of a word or lemma into an orthographic hand-arm-motor form: O-prod, Figure 1). However, this part of the model has not yet been implemented.

From the point of view of speech acquisition, the “large processing loop” described above can also be seen as a “large learning loop.” Let us imagine the model as a child, who sees a ball for example (activation of C_perc, C_cog, C_prod for the concept “ball”) and now wants to name this object (activation of L_prod and P_prod for the word “ball”). For this purpose, the child will draw the caregiver's attention to the ball in a specific process scenario, here called “word learning.” Then, by looking at it, the child will motivate the caregiver to pronounce the associated word “ball” (triangulation process: child looks at the ball, points to the ball, looks at the caregiver). This process scenario is implemented in the model in the following way: The concept “ball” is activated in a preliminary neuronal specification at the cognitive level (C_cog). The child then tries to realize the word (the syllable) by activating preliminary neural specifications of motor plans (M_prod -> S_perc and M_prod -> A_perc due to the self-perception of what has been said). Based on the target word uttered by the caregiver, the child already has an idea of the auditory form of the word (A_perc). In an iteration process, the motor form of the word or syllable (M_prod) and the speech movement units (gestures G_mot) involved in this syllable are produced now and are modified and optimized until the caregiver accepts the acoustic correlate of the word production done by the child after several attempts. The accepted speech items are then stored in the mental lexicon and in the mental syllabary (see also Kröger and Bekolay, 2019, p. 71ff; Kröger et al., 2019 for the formulation of this learning scenario in a connectionist approach).

In the course of language acquisition, the model acquires more and more syllables, words and phrases and thus also recognizes grammatical structures and will thus also build up knowledge at the lemma level (Figure 1). In addition, through linguistic as well as non-linguistic interactions with the environment, the child will also build up a world knowledge and thus learn a lot of semantic concepts and also build up a set of relationships between concepts (see the mental lexicon and world knowledge, Figure 1). Over time, the child will also be able to expand the set of phonological forms and derive syllabic-phonological structure rules (phonotactic rules) from the large number of syllables learned (see the set of all phonological forms in the mental lexicon and in the mental syllabary; Figure 1). The same applies to the amount of motor plans and to the amount of speech movement units. Thus, in the course of language acquisition, all areas of the mental lexicon and mental syllabary are built up. Stored items can be activated both on the production side and on the perception side on each level of the production model (i.e., the concept level, the lemma level, the phonological form level, the level of the sensorimotor plans, and the level of speech movement units).

All levels of our knowledge and skill repository have been addressed in the language acquisition process. Thus, a defined set of items has been learned and saved for each of these levels. These items are modeled by sets of S-pointers and S-pointer-networks, which can be defined for each level of the model. Each of these levels is linked from neural state buffers on the production side to neural state buffers on the perception side. These buffers can each contain an item or a sequence of items as a neural activity pattern for a short period of time in order to be able to process a word in the top-down manner defined by the model. The arrows of the model in Figure 1 indicate that a neuronal activation occurring in a state buffer activates the next state belonging to the same syllable or word in the subsequent buffer of the next higher level (on the perception side) or the next lower level (on the production side). Thus, the thick brown arrows in Figure 1 describe the normal course of the neuronal transformations within the production and perception channels. While at the higher levels, the same types of representations occur at each level, we have to cope with different types of representation at the lower process levels. Here we must differentiate between sensory and motor specifications of the motor plans as well as the speech movement units. It can be assumed that motor plans on the sensory side correspond to auditory forms of whole syllables, while speech movement units or sound-related small production units correspond more with somatosensory information (tactile and proprioceptive movement information; see Hickok, 2012).

**Figure 2:**
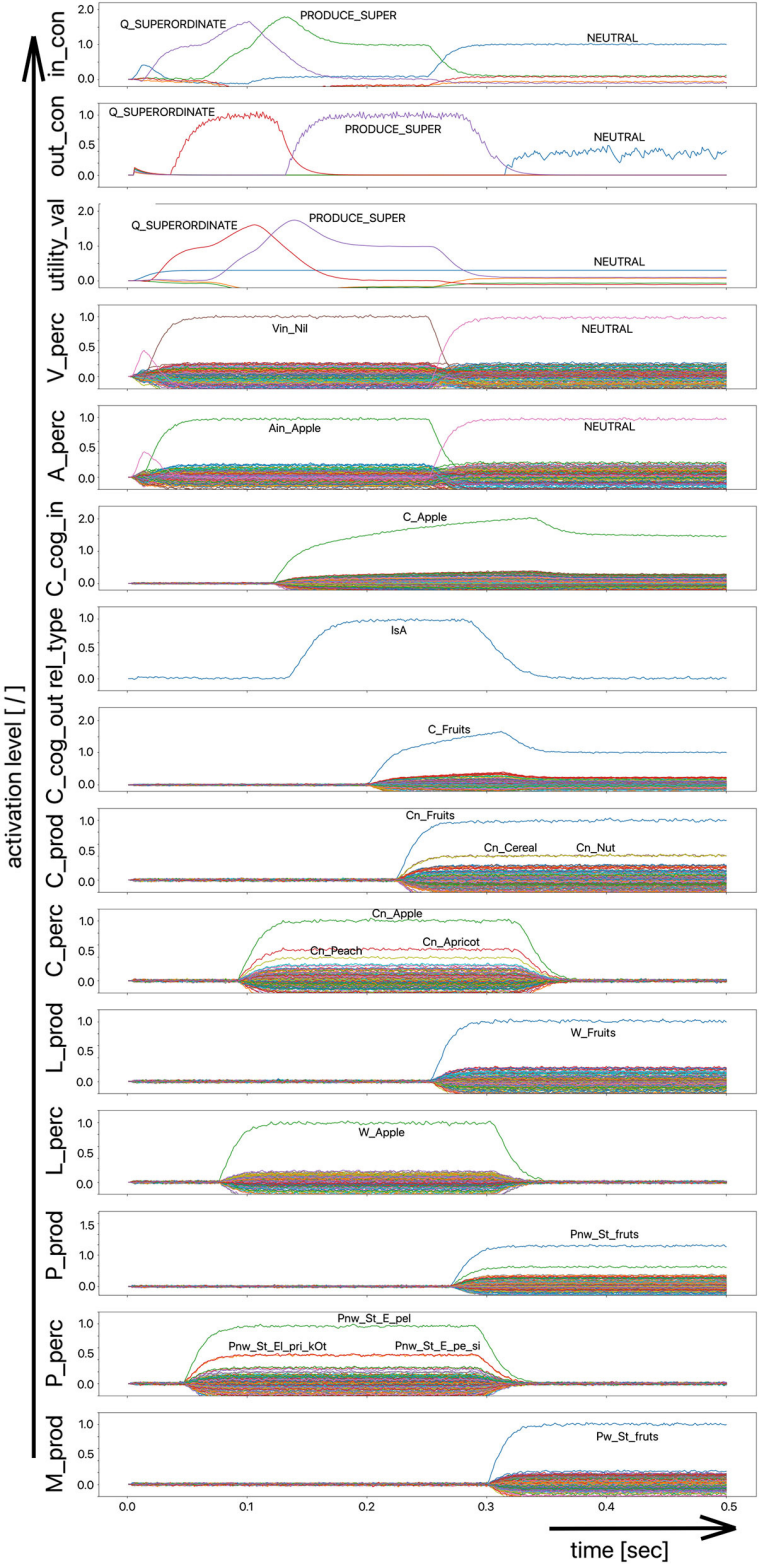
Decoded neural information in the neural state buffers of different model levels on the perception and production side by displaying activation strength of states (y-axis; the value one indicates an activation level of 100% for a specific neural state) over time (x-axis in sec). The task defining the process scenario is word perception (here of the word “apple”), word comprehension, and word processing (here of the word “apple is a”) and word production of the result of cognitive processing (here: the word “fruits”). State buffers and there activation patterns from above: input control activity for the process scenario (in_con); generated actions of the control module (out_con); utility values for the selection of actions [see “action selection” processes in Stewart and Eliasmith (2014)]; visual input (arrow under V_perc); auditory input (arrow under A_perc); Concept input for the cognitive processing module (C_cog_in); Relation type input for cognitive processing (rel_type); cognitive output from the cognitive processing module (C_cog_out). Remaining part of the figure: state buffer activations at the concept level (states are named: C_. and Cn_.), lemma level (L_.) and phonological form level (P_ and Pn_.) on the production and perception side (small “n” indicates S-pointers which are part of a S-Pointer network; see text; small “w” indicates “word” in contrast to sub-word units like syllables). On bottom: state buffer activation patterns of the motor plan on the production side (see text). Activation patterns are decoded in form of S-pointer-amplitudes for each speech item at each model level. The standard S-pointer amplitude (i.e., the activation strength of the appropriate neural state) in these “similarity plots” (cf. Eliasmith, 2013) is one (unity length). It can be assumed that a neural representation is activated sufficiently in a state buffer, if the amplitude of the associated S-pointer is higher than 0.7. The display of activation levels is limited to value of two (see C_cog_in and C_cog_out) for all figures, because no new information is given if the activation level of a state is higher than “full activation” (i.e., one). Activation levels higher than one sometimes occur due to a building up of neural energy in a memory buffer, if information is transferred that memory (see figures below).

Since the “large processing loop” which can be identified from Figure 1 underlies the hierarchical structure of language production and thus the idea of “sequential processing” of neural signals, it should also be remembered that every neural state activated in a state buffer is activated for longer time periods on each side (production or perception) on each level (concept level down to the levels of sensorimotor representations). This becomes clear from the visualization of the neuronal activity of each state buffer over the entire course of a combined word perception, word comprehension, and word production scenario (Figure 2). The sequence of activations on different model levels and their overlapping activation time intervals can be recognized here for each S-pointer activated in the state buffers. The model thus clearly shows the flow of information during speech processing while also indicating the temporal overlap of activations at different model levels.

Due to the priming of the test person (the model) with regard to the task to be executed (here: word recognition and word comprehension based on auditory inputs (here the word “apple”) with identification of a superordinate term for this word (here: “fruits”), the input signal for the control module first defines a time interval for recognizing the input word for later determination of the superordinate term (Q_SUPERORDINATE). The action to activate the generic superordinate term (PRODUCE_SUPER) is then activated. The auditory input activity extends over almost both time intervals and activates the lemma and the concept of the word “apple” within the perception channel. At the level of cognitive processing, the S-pointer for the semantic relation “is a” is then activated in the state buffer (rel_type; not indicted in Figure 1). Within the cognitive processing module, a binding process between “apple” and “is a” takes place, which subsequently leads to the activation of the concept “fruits.” This result is then passed on to the output state buffer of the cognitive processing module. The word “fruits” is then activated and implemented as soon as it passes to the production channel. No further action is needed from the process model for word activation and word processing within the production channel down to the motor plan state. It can be seen that between the activation of the auditory word input and the activation of the motor plan output, there is a time delay of ~250 ms.

It should also be pointed out that the coupling between the currently activated state of a word and the mental lexicon can also be recognized very well from Figure 2. For example, the phonological similarity relationships can be recognized from the co-activation of phonologically similar words (here: “apathy” and “aprikot” for the activated word “apple”) in the phonological buffer on the perception side. Similar relationships can be recognized for the semantically similar words at the concept level on the perception side (here: “apricot” and “peach” for the primarily activated word “apple”).

Furthermore, it should be stated here that activation levels of neural states are visible in the similarity plots, or plots indicating the activated neural states in a specific neural buffer (see Figure 2 ff). Here normal activation levels are indicated by S-pointer amplitudes of about 1 (unit length S-pointers). This reflects 100% activation of a neural state. We will see later, that already an activation below 70% (amplitude of 0.7 in the similarity plots for a specific S-pointer) already may lead to erroneous processing because activation amplitudes normally decrease to a certain degree with each neural forwarding and neural processing step. Processing buffers like associative memories normally cannot process states indicating activations below a level of 0.3 in the similarity plots (i.e., a state activation below 30%). Thus, we define this 30%-level as threshold activation level for further processing of a neural state.

Moreover, it should be mentioned that neural memories in the form of recurrent neural state buffers can exhibit state activities represented by S-pointers with a length above one, because during the signal input time interval the recurrent neural connections together with the neural input connections amplify a neural state strongly. That automatically stops if the input signal time interval ends, because the recurrent neural connections from now on only serve to hold the input signal at a particular intensity level, represented by a S-pointer length of about one.

At this point of the paper we want to address an important question: How do concepts from the NEF-SPA approach inform the structure of our actual speech production model? This question can be answered by addressing three main points:

(i) The NEF-SPA clearly separates knowledge storage and (dynamic) neural processing. Knowledge is stored in the form of sets of S-Pointer and of S-Pointer networks. These sets or networks are fixed in the model before the simulations start. Each S-Pointer represents a state (e.g., a word or an executable action). The neural realization of an S-Pointer corresponds to a particular pre-defined activity pattern of the neurons in that buffer.
(ii) Only a few building blocks (buffers, memories, connections) are required for neural processing and thus for developing a speech production model if the NEF-SPA approach is used. Buffers allow neuronal states to be activated at certain time intervals at certain locations within the overall neural network. These states can either only be activated as long as they are carried by the buffer input, or they can be held in a buffer for a short period of time if this buffer is additionally equipped with recurrent neuronal connections (short-term memory). This concept of neural engineering is sufficient to model lexical access in language production.
(iii) Neural connections can be realized in the NEF-SPA approach by direct connections between buffers if the neuronal information is only to be passed on. Neural connections that transform neural states, for example through the conversion of an S-pointer A into an S-pointer B, always requires the interposition of an associative memory, which contains exactly the knowledge of how each S-pointer must be transformed. Associative memories enable the realization of different model levels in our lexicon model (concept level, lemma level, phonological form level, engine plan level) as part of the entire speech production model.
(iv) In the NEF-SPA, in addition to the neural transformation of S-pointers done by means of associative memories, an additional neural transformation of S-pointers by defining binding and/or unbinding processes can also be implemented. In our modeling approach for word production, binding and unbinding processes are only suitable for processing S-pointers within an S-pointer network, and to thereby store short-term relations in short-term memories (e.g., binding a specific object with a specific color currently of interest: “(this is a) blue ball” vs. “(this is a) red ball”) while associative memories process knowledge stored in long-term memories in order to unfold stored relations between objects or concepts like e.g., “an apple” (object) “is a” (relation)? “fruit” (object). The neural mechanism of binding and unbinding can be realized in the NEF-SPA approach like the neuronal mechanism of simple S-pointer transformation by means of defining specific binding and unbinding buffers. In our model, this concept of the NEF-SPA is only used at the level of cognitive word processing.

It should be noted that this minimal set of concepts prescribed by the NEF-SPA is sufficient to adequately model the complex neuronal mechanisms of word production. Through this minimalism, a clear model of word production can be created that is capable of simulating normal as well as disrupted word production. The reduction to a few neural storage and processing principles is therefore the key to creating the model described in this paper.

### Different Process Scenarios

**Figure 3:**
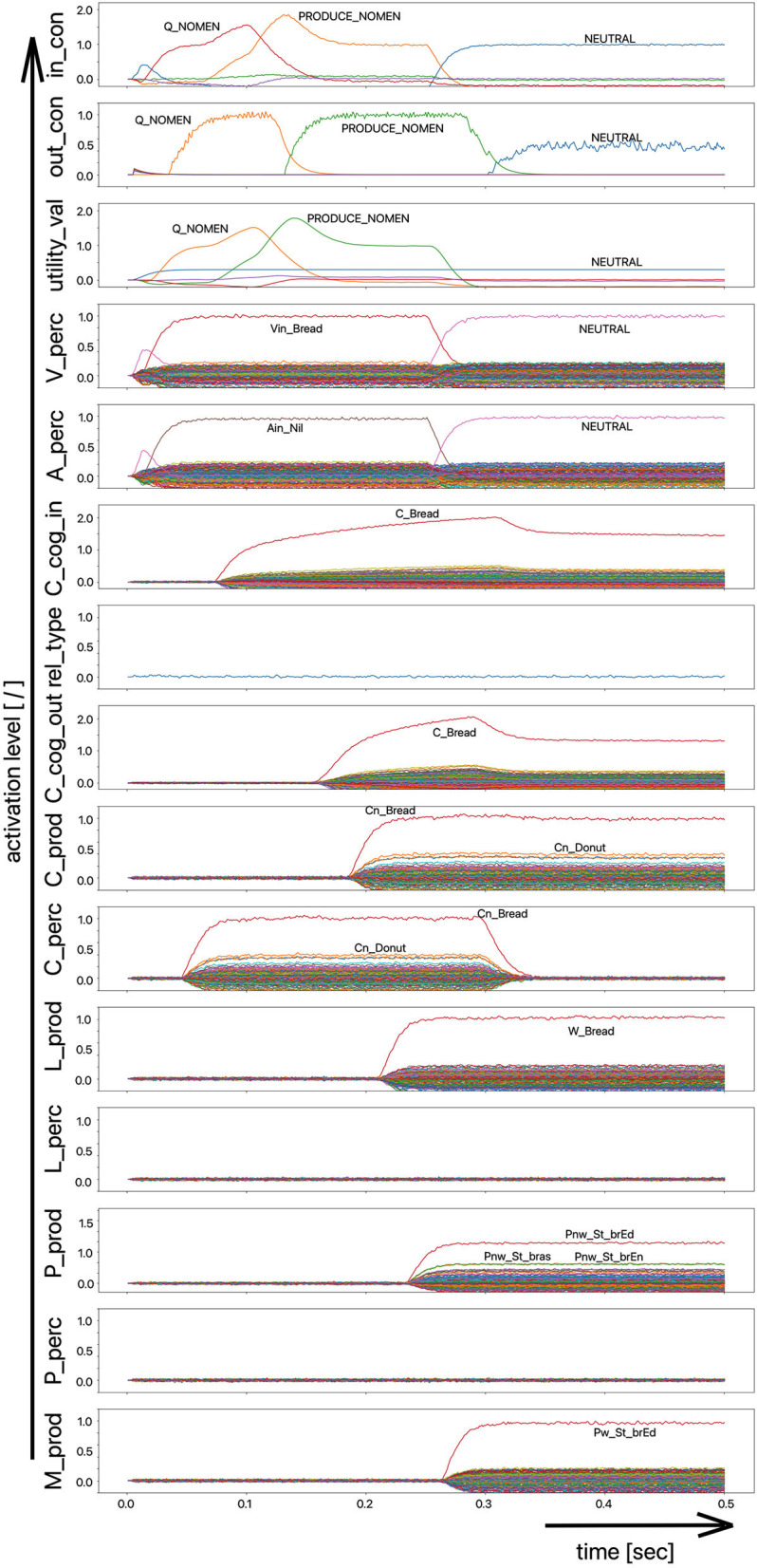
Decoded neural information in the neural state buffers at different model levels on the perception and production side. For a description of the individual buffers, see Figure 2. The process scenario is that of a picture naming task (see text).

**Figure 4:**
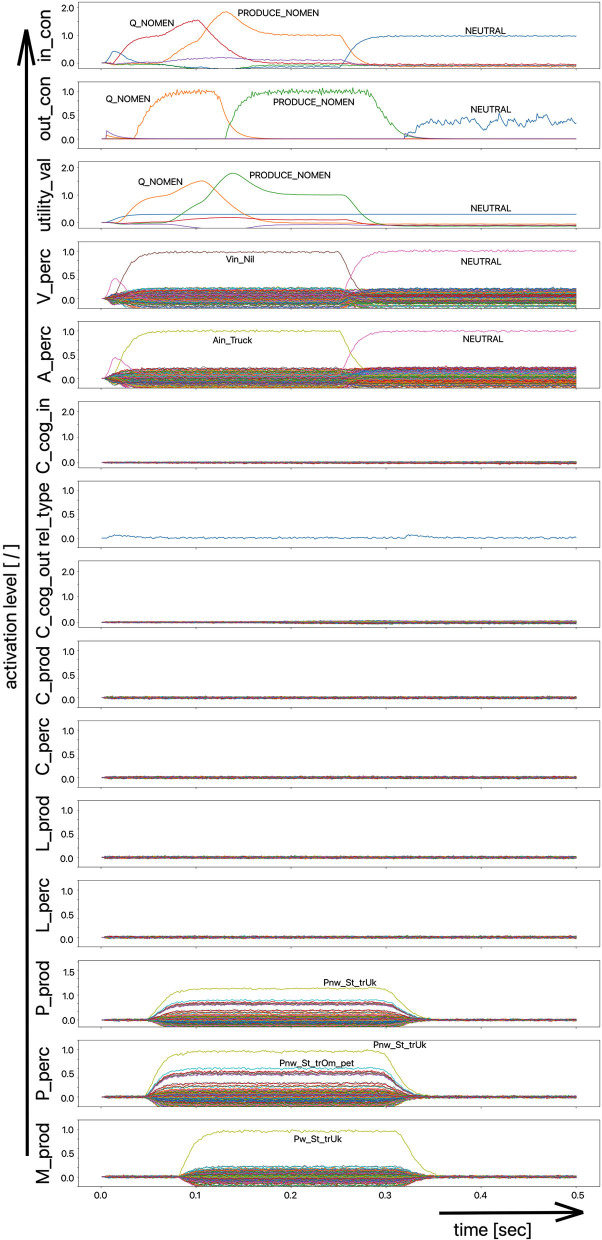
Decoded neural information in the neural state buffers at different levels of the model on the perception and production side. For the description of all state buffers, see Figure 2. The process scenario is that of a word or logatome repetition task (see text).

The tasks to be performed by a test person define the simulation scenarios to be performed by the model. In the context of this paper, we simulated three different tasks or process scenarios:

(i) A picture naming task wherein the item to be named is presented visually as the main part of a picture. It is the task of the test subject or model to produce the associated word. An example of the sequence of activations of state buffers for this process scenario is given in Figure 3. Here the word “bread” is offered as a visual stimulus (visual_in) and recognized as such (C_perc). The word is passed through on the cognitive processing level without further processing steps (C_cog_in -> C_cog_out) and then passes through the production channel down to the motor plan state buffer.
(ii) A word comprehension task wherein a term is offered auditorily (e.g., by the test supervisor) and it is the task of the subject or model to find and produce a generic term or superordinate describing the word (see Figure 2: perception of the word “apple” and expression of the generic term or superordinate term “fruits”).
(iii) A word (logatome) repetition task wherein a syllable or a combination of syllables is offered auditorily. However, this should not be a word which occurs in the subject's mental lexicon, but rather a meaningless word or logatome. The subject is then forced to imitate the word on the phonetic-phonological level. We simulate this in the model by cutting the connection between the phonological state buffer and the lemma state buffer in the perception channel. Thus, the model cannot recognize the word, even if it is not a logatome, and cannot transfer it to the production channel via the concept level. Rather, the model is forced to use a direct or “shortcut” neural association between the two state memories on the phonological level, namely from the perception side (P_perc) to the production side (P_prod; see also the thin brown arrows on that level in Figure 1). The activation pattern for a perceptually offered example syllable (here: “truck”) is shown in Figure 4. There are no neuronal activations above the phonological level, because the mental lexicon is disconnected. The phonological form is thus transferred directly from the perception side to the production side at the phonological level. However, the activation of the motor plan, which begins about 500 ms after the start of the auditory stimulus, does not mean that articulation is already beginning at this point in time. The resulting activation of the sequence of speech movement units (G_mot) in the primary motor area of the cerebral cortex and the consequent initiation of articulation movements is not shown in Figure 4, since this part of the model has not yet been implemented. A first version of this motor part of the model part is discussed in Kröger et al. (2016a).

### On the Influence of Feedback Mechanisms

In addition to the “large processing loop” (indicated by thick brown arrows in Figure 1), there are a number of smaller feedback loops, indicated by thin brown arrows in Figure 1. The associated feedback mechanisms play an important role both during speech acquisition and later during speech production. Three more feedback loops are discussed here.

First, there is the “motor plan feedback loop” (S_perc and A_perc -> M_prod, and further down on the production side toward articulation; Figure 1): This loop plays an important role in learning motor plans. In the babbling phase of speech acquisition, motor-auditory and motor-somatosensory relationships are initially perceived based on randomly produced speech articulator movements. This basic knowledge is stored in the lower levels of the mental syllabary as well (Kröger and Bekolay, 2019, p. 71ff). This basic knowledge of sensorimotor relationships allows the subject to successfully imitate speech items (Kröger and Bekolay, 2019). This subsequently allows the subject to build up the higher levels of the mental syllabary, i.e., sensorimotor representations of all frequent syllables. The syllabic motor plans and the associated auditory and somatosensory representations of each syllable are then stored. After the mental syllabary has been built up, the subject is able to call up motor plans for the learned syllables immediately and to implement them correctly in the articulatory processes. In addition, the subject is now able to compare the auditory and somatosensory states resulting from a current articulation with the already learned auditory and somatosensory states for the syllable just realized.

If there are discrepancies between the sensory results of a syllable that has just been realized and the stored sensory patterns for the correct realization of a learned syllable that has just been produced, then the motor plan of that syllable is modified. This change is such that the new sensory patterns come closer to the stored sensory pattern (i.e., the sensory target state of the already learned syllable) than the previous realization of the syllable (see Guenther, 2006; Guenther et al., 2006). Accordingly, the motor plan feedback loop is not only required in speech acquisition for the construction of a repository for correct sensorimotor relations within the lower levels of the mental syllabary (Figure 1), but also afterwards for the continuous verification of the correct realization of all syllable productions by becoming aware of discrepancies between current ones and stored sensory target states.

However, this feedback loop triggers actions via the control module if the correction of a word or a syllable is needed. In the event of a discrepancy between the sensory states of syllables that have just been realized and their learned sensory target states, the correction process is initiated via the basal ganglia and thalamus, but only after finishing the incorrect realization of the syllable. In the event of such a discrepancy, the motor plan of the syllable is modified and checked again during the new articulation process initiated by the control module. The new motor plan should bring the sensory target and the sensory state of the actual realization closer together. In the mental syllabary, due to the multitude of production attempts already done by the child during speech acquisition, knowledge is stored concerning the direction in which the parameters of the motor plans and of gestures have to be changed. This is done in order to bring the sensory target closer to the already learned target (see the definition of the “error maps” according to Guenther et al., 2006).

Second, there is the “feedback loop for speech movement units” (also called: “gesture feedback loop:” S_perc -> G_mot, and further down on the production side toward articulation). The motor plan of a syllable defines the types of speech gestures and specifies the number of speech gestures per syllable and their temporal coordination within the syllable. The speech gestures or speech movement units themselves contain the information for the implementation of target- or goal-directed vocal and consonant articulation movements (Kröger and Bekolay, 2019, p. 17ff). Each speech movement unit defines a targeted movement of one or more articulators (e.g., lower jaw, lower lip, and upper lip in case of the realization of a bilabial closure). It has already been emphasized that in addition to the use of auditory information, speech movement units are primarily controlled by means of somatosensory feedback information. For vocalic gestures (e.g., shaping the vocal tract to articulate an /a/), this somatosensory movement and target information can be in the form of a proprioceptive target: (i) adjustment of the muscular tension and stretching for the tongue body and (ii) adjustment of the joint angles for example of the temporomandibular joint when lowering the tongue body and opening the mouth for producing the vowel /a/. For high vowels (e.g., /i/ or /u/) there will additionally be tactile information about the contact area and contact strength of the tongue edges with the side edges of the hard and soft palate. In the case of the consonant articulation, the sensory target is mainly in the form of tactile information, such as the location and size of the contact surface of the tip of the tongue with the hard palate when an apical closure such as /t/ or an apical constriction such as /s/ is produced. While acoustic information is more complex can only be processed slowly, somatosensory feedback information can be processed very quickly, such that the gesture feedback loop can even be used for online corrections of the articulation movements of gestures (Parrell and Houde, 2019). The correction information for these simple, goal-directed articulation movements, which in particular result in the realization of individual speech sounds, is therefore also partially stored in the cerebellum (Hickok, 2012). The part of the gesture feedback loop that is controlled by the cerebellum can therefore do online movement corrections. The auditory part of the sensory feedback information is only partly used to adjust the parameters of (mainly vocalic) speech movement units. Basically, the acoustic information serves to check the correct temporal coordination of all speech gestures involved in the production of a syllable, a word, or an utterance.

Third, there is the “internal (cognitive) feedback loop” (P_prod -> P_perc, and further up within the perception channel; Figure 1). It has been shown that internal perceptions of the phonological form, the lemma, and the concept of the word to be produced occur earlier than the motor realization of that word. So even during the production of a word, it can be perceived “internally” before the articulation of the word starts (Postma, 2000). Therefore, it is assumed that there is a feedback shortcut on the phonological level from the production to the perception side (i.e., in the opposite direction from the short cut necessary for logatome repetition). This feedback enables an early correction of word production if, for example, an incorrect word selection has already taken place in the production channel at the lemma level. It can be assumed that this internal feedback loop is able to compare the concept, lemma, and phonological forms of a word under production between the perception channel (i.e., for the intended word) and the production channel (i.e., for the produced form). If there is a discrepancy between these two forms, the ongoing production of the word can be interrupted so that a new (correct) production attempt can start, starting with a new selection process for that concept. Both the termination of the current (wrong) production and the start of a new (possibly correct) production is carried out via the control module. Such a “stop” of the wrong production can be evoked, in particular during a picture naming task with auditory interspersed distractor words (Slevc and Ferreira, 2006).

### Modeling Speech Disorders and Speech Errors

Since we want to model the effects of defined speech disorders and of speech errors that occur spontaneously in normal subjects in this paper, a brief overview of speech disorders and methods of triggering speech errors is given here. All types of speech disorders and speech errors mentioned here were simulated in this study.

According to the model of the mental lexicon (Levelt et al., 1999), six different types of aphasias can be distinguished (Roelofs, 2014). In Broca vs. Wernicke aphasia it is assumed that the phonological state buffers on the production side vs. the perception side are neuronally disturbed. In the case of transcortical motor vs. transcortical sensory aphasia, it is assumed that the associative memories between the phonological form buffer and the lemma buffer are disturbed on the production side vs. the perception side. Mixed aphasia assumes that the associative memories between the lemma and concept buffers are disturbed on both the production and the perception side. Conduction aphasia assumes that the neuronal connections between the phonological state buffers reaching from the perception to the production side are disturbed. Roelofs (2014) presents a computer simulation of the resulting symptoms in picture naming, word comprehension, and logatome repetition tasks by implementing these neural disruptions in different buffers or memories at different levels of the production model. These types of neural disruptions can be modeled in our approach by ablating a percentage of neurons in specific state buffers as well as in associative memories, which in the second case leads to a disruption of neural connections between state buffers. For modeling a healthy person, the percentage of ablated neurons is zero. The degree of a neural dysfunction and thus the severity of a disorder increases with increasing percentages of neural ablation in specific buffers.

Other typical neurogenic speech disorders are apraxia of speech and the dysarthrias. Apraxia of speech is defined as a motor planning disorder where motor plans cannot be learned, stored, or retrieved correctly. Dysarthrias are defined as a set of speech disorders stemming from disruptions of the neural motor pathways and the muscular systems of the speech articulators. Thus, the correct execution of activated motor plans is not possible. Modeling the resulting speech behavior and the symptoms of these neurogenic speech disorders is difficult. Kearney and Guenther (2019) discuss the localization of these speech disorders in the module structure of their model, but do not yet provide any simulation examples.

Higher level speech errors can be triggered, for example, by using the experimental paradigm described by Slevc and Ferreira (2006). Distractor words are interspersed during a picture naming task. The subject is asked to either stop the production of a word in different runs of the test (“stop-trial”) when she/he hears the distractor word, or to try to continue word production despite the occurrence of the distractor word (“go-trial”). It has been shown that those distractor words that are phonologically similar to the target word to be produced are ineffective. Stopping word production is therefore more likely the less similar the distractor word is in comparison to the target word to be produced. In particular, semantically similar distractor words had the same effect as completely dissimilar distractor words, while the distractor word is more ineffective in stopping picture naming if it is phonologically similar. A first computer simulation of this test scenario was developed by Kröger et al. (2016b). The behavioral effects could be modeled with regard to the effect of dissimilar and phonologically or semantically similar distractor words. In particular, in this paper examines which level of the internal feedback loop triggers the effect.

It has been shown that speech errors occur frequently for speakers suffering from speech disorders, for example when lexical access is disturbed or when the motor planning or motor execution levels are disturbed (Liss, 1998). In this case, speech errors are not spontaneous like in the case of healthy speakers, but rather are frequently occurring errors, which often fluctuate with regard to the type of error. In an experiment in this paper we simulate lexical retrieval disorders and the resulting speech errors that occur in that case. A study introducing the basic test scenario for this test is introduced by Stille et al. (2019).

## Method: Description of Simulation Experiments

### Experiment 1: Neuronal Dysfunctions at Different Levels of the Model Due to the Modeling of Different Forms of Aphasia

Three simulation scenarios were implemented: a picture naming task, a word comprehension task, and a logatome repetition task. Each simulation scenario was implemented for all 18 target words for the picture naming task (see Table 1). These 18 target words as well as 18 phonetically similar, 18 semantically similar, 18 semantically and phonetically similar and 18 semantically and phonologically dissimilar words were stored in the mental lexicon at the concept level, the lemma level, and the level of the phonological forms (see Table 1). Similarity relations were realized on a semantic and phonological level. To define the semantic similarity relations, 18 generic terms or superordinate words were defined and stored in the mental lexicon. To define the phonological similarity relations, phonological sound sequences were defined and also fixed as logatomes. For the transcription of all phonological forms for each word, see Kröger et al. (2016b). It should be stated here that the limitation of a vocabulary to just about 100 words does not limit the generality of the results (cf. Roelofs, 2014, p. 37).

**Table 1:** Words and sound combinations anchored in the mental lexicon for all three simulation experiments of this study.

**Target word**	**Semantically similar word**	**Phonologically similar word**	**Phonologically and semantically similar word**	**Dissimilar word**	**Semantically superordinate item (semantic cues)**	**Similar sound segments (phonological cues)**
Apple	Peach	Apathy	Apricot	Couch	Fruits	/Ep/
Basket	Crib	Ban	Bag	Thirst	Bin	/bE/
Bee	Spider	Beacon	Beetle	Flag	Crawler	/bi/
Bread	Donut	Brick	Bran	Nail	Cereal	/br/
Camel	Pig	Cash	Calf	Bucket	clovenHooved	/kE/
Carrot	Spinach	Cast	Cabbage	Evening	Veg	/kE/
Duck	Raven	Sub	Dove	Brass	Bird	/da/
Elephant	Moose	Elm	Elk	Stripe	HornAnimal	/El/
Fly	Moth	Flu	Flea	Rake	Bluebottle	/fl/
Lamp	Candle	Landing	Lantern	Package	LightSource	/lE/
Peanut	Almond	Piano	Pecan	Dress	Nut	/pi/
Rabbit	Beaver	Raft	Rat	Coffee	Rodent	/rE/
Snake	Eel	Snack	Snail	Fire	Invertebrate	/snE/
Spoon	Ladle	Sparkle	Spatula	Cable	Lifter	/sp/
Squirrel	Mole	Skate	Skunk	Chain	HairySkin	/sk/
Train	Bus	Trophy	Trolley	Fox	PublicTrans	/tr/
Truck	Jeep	Trap	Tractor	Celery	UtilityVehicle	/tr/
Trumpet	Horn	Traffic	Trombone	Corner	BrassWind	/tr/

*The semantic cues are also anchored in the mental lexicon as meaningful units. These terms (superordinate words) are not necessarily real words of the target language and in addition need not necessarily have to be correct in an overall sense, because the mental lexicon is subject-specific and represents world knowledge of an individual. The phonological sound segment sequences (phonological cues; last column of the table) can be interpreted as logatomes. They have no equivalents at the lemma and concept level. The phonological transcription symbols correspond to the SAMPA standard (SAMPA, 2005)*.

In the case of the logatome repetition task, the neural association between the state buffer of the phonological forms and the state buffer of the lemmas was separated within the perception channel, so that the target words are interpreted and processed by the model as meaningless syllable sequences (logatomes). Example activations for all three simulation scenarios have already been given in the theory section of this paper (Figures 2–4).

### Experiment 2: Picture Naming Task With Distractor Words in a Halt Scenario

**Figure 5:**
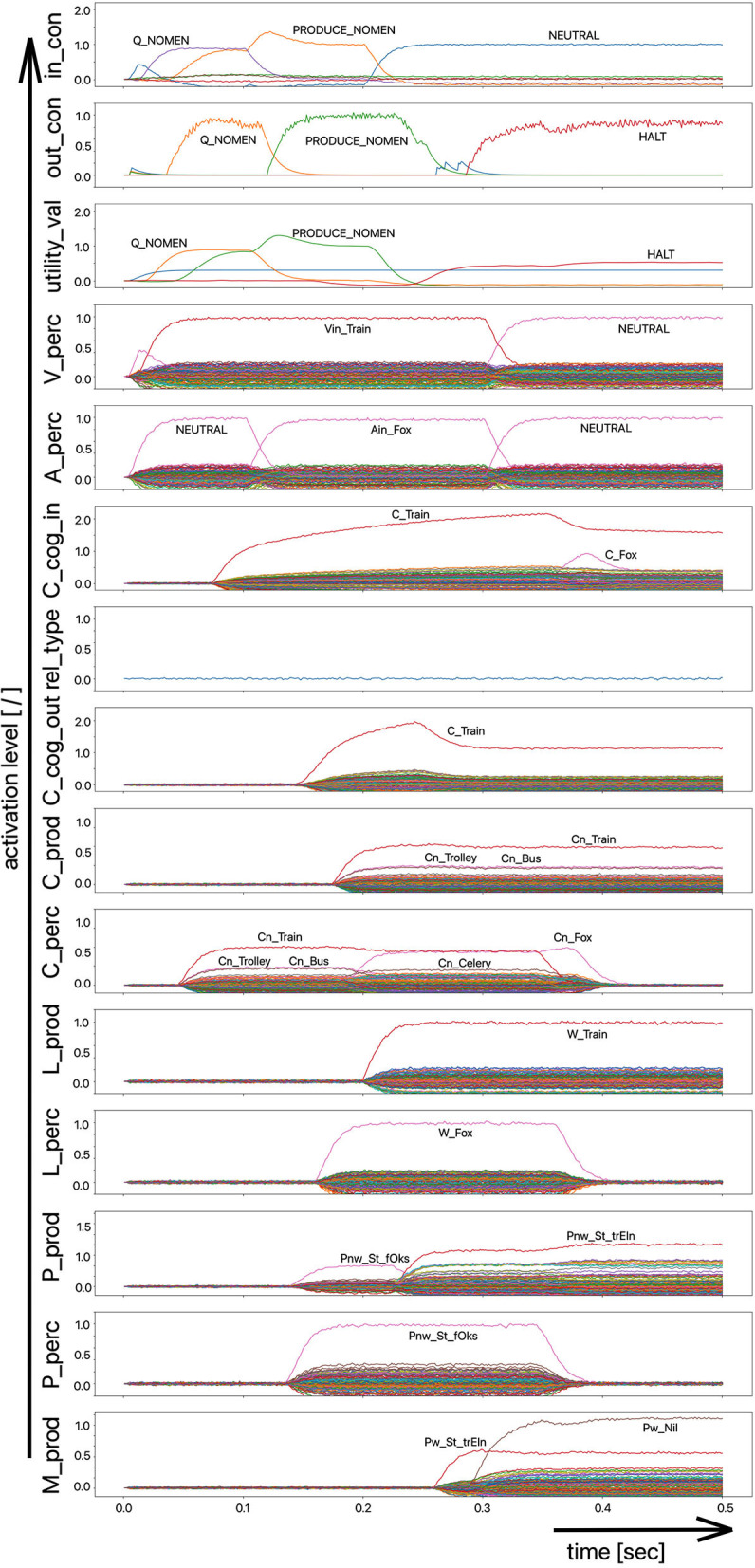
Decoded neural information occurring in the neural state buffers at different model levels on the perception and production side. For the description of the individual state buffers, see Figure 2. The process scenario is a picture naming task (target word here: “train”) with an acoustically interspersed distractor word (here: the phonologically and semantically dissimilar word: “fox”). See text for further explanations.

**Figure 6:**
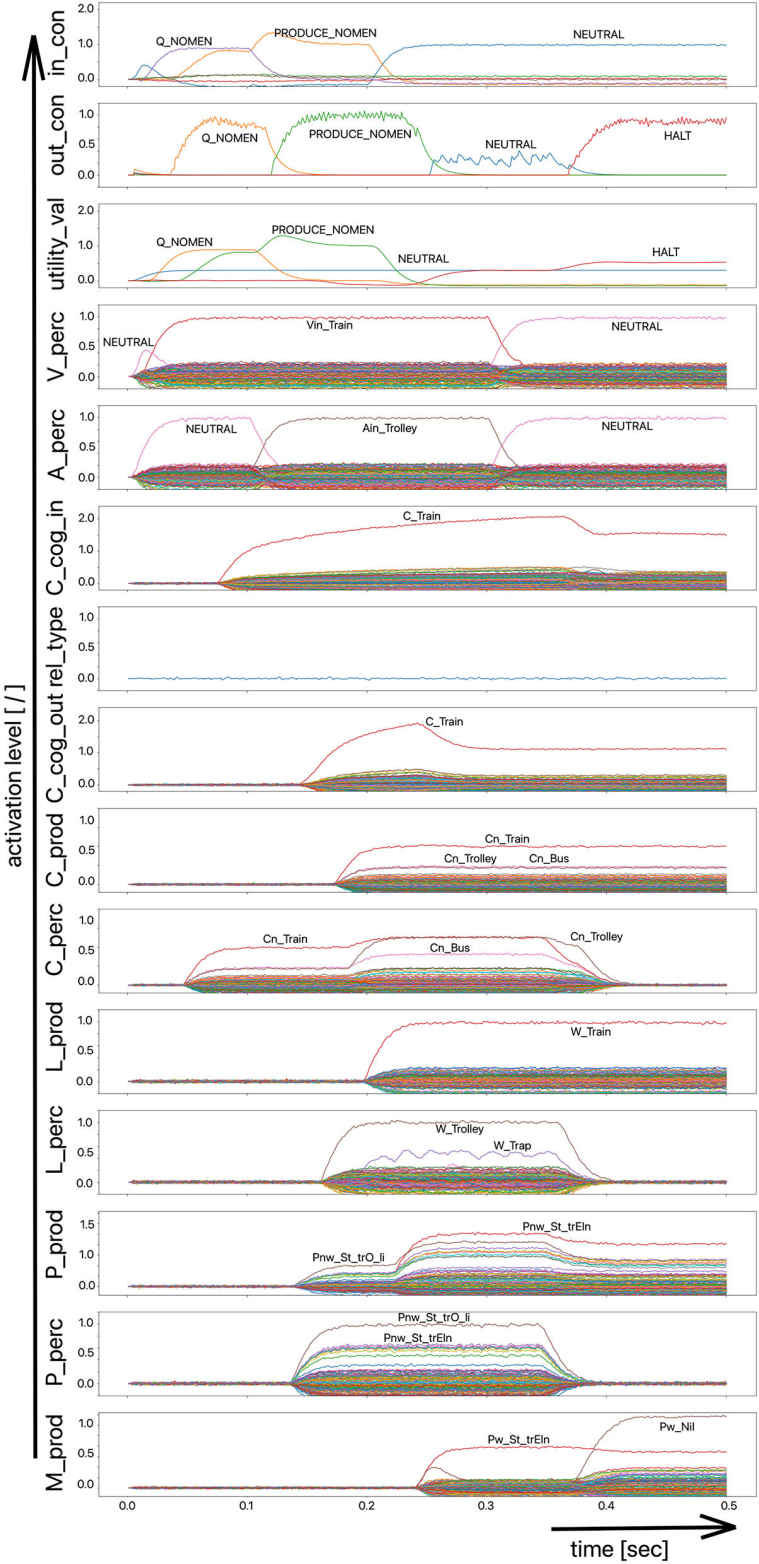
Decoded neural information in the neural state buffers at different model levels on the perception and production side. For a description of the individual state buffers, see Figure 2. The process scenario is a picture naming task (target word here: “train”) with an acoustically interspersed word (here: phonologically and semantically similar word: “trolley”). See text for further explanations.

The scenario simulated in this experiment is based on a picture naming task in which a distractor word is acoustically interspersed during the production of the target word (Slevc and Ferreira, 2006). In our implementation of this experiment, the distractor word, an auditory stimulus, is interspersed 50 ms after the visual stimulus containing the target word is presented (Figures 5, 6). Although the production of the target word is permitted, a HALT-signal (S-Pointer HALT, see Figures 5, 6) is generated based on the evaluation of the strength of the activation difference (i) in the phonological state buffers alone or (ii) in the phonological and semantic state buffers of the production and perception sides of the model. If the absolute value of this difference is too high, the HALT action is triggered. Thus, the HALT action occurs if the difference in the activations between the production and the perception side exceeds a certain threshold. This activation of the HALT action is activated in the control module of our model (out_con; see Figures 5, 6). In our simulations, however, this action only causes the activation of a motor-level S-pointer “Nil” in the motor plan buffer which overlays the motor plan S-pointers of the target word (herein Figures 5, 6: “Pw_St_traIn,” which points on the motor realization of the word “train”).

If the distractor word is phonologically dissimilar to the target word, a strong and only slightly (about 20–40 ms) delayed Nil signal occurs in the motor plan buffer (Figure 5). However, if the distractor word is phonologically similar, a much weaker and more time-delayed Nil-signal (delay time ~100 ms) occurs in the motor plan state buffer (Figure 6).

### Experiment 3: Phonological and Semantic Retrieval Aids

**Figure 7:**
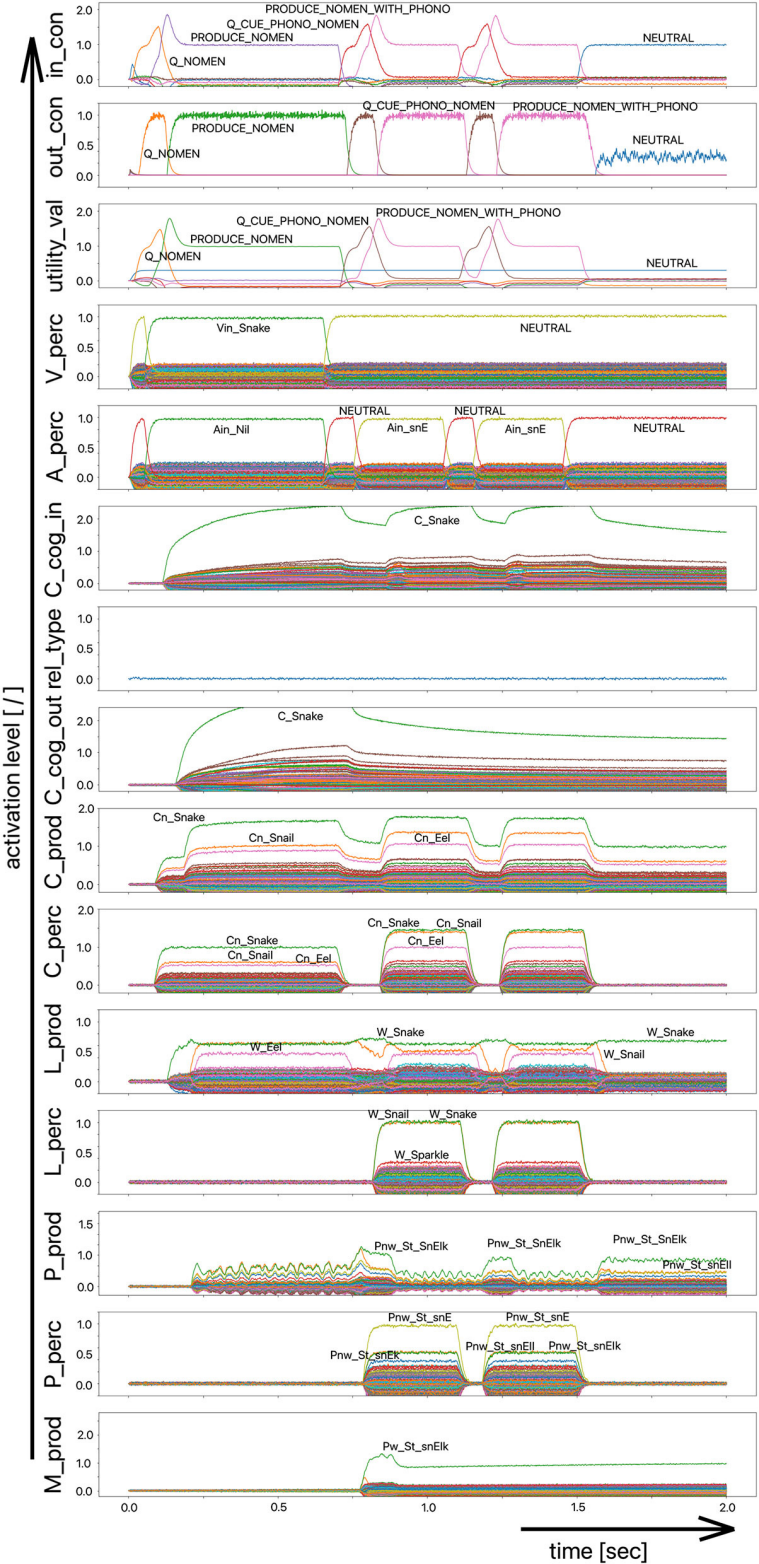
Decoded neural information in the neural state buffers of different model levels on the perception and production side. For the description of the individual buffers see Figure 2. The process scenario up to 750 ms is a picture naming task (target word here: “snake”). After 750 ms, the test supervisor provides an additional phonological cue via the auditory channel [“(the target word begins with) /snE/”]. In this simulation example, this cue leads to the full activation of the correct word at the motor plan level.

As part of the “linguistic test for lexical storage and lexical retrieval for Standard German” (WWT 6–10, Glück, 2011), we are using the picture naming task for the 18 target words introduced above (see Table 1) in this simulation experiment. In a pilot experiment, the associative connections between the concept and lemma state buffers on the production side were dampened by decreasing the output amplitude of the neural signals traveling through this connection, so that the correct and full activation of a lemma even in the case of a correct and complete activation of the concept leads to lemma activations with low amplitude (i.e., activation strength of <70% of normal state activity in M_prod). From Figure 7 we can see that the activation of the target word “snake” at the lemma level in the production channel leads to the activation of three lemmata, i.e., “snake,” “snail,” and “eel,” at the same time and both S-pointers have a low activation level.

**Figure 8:**
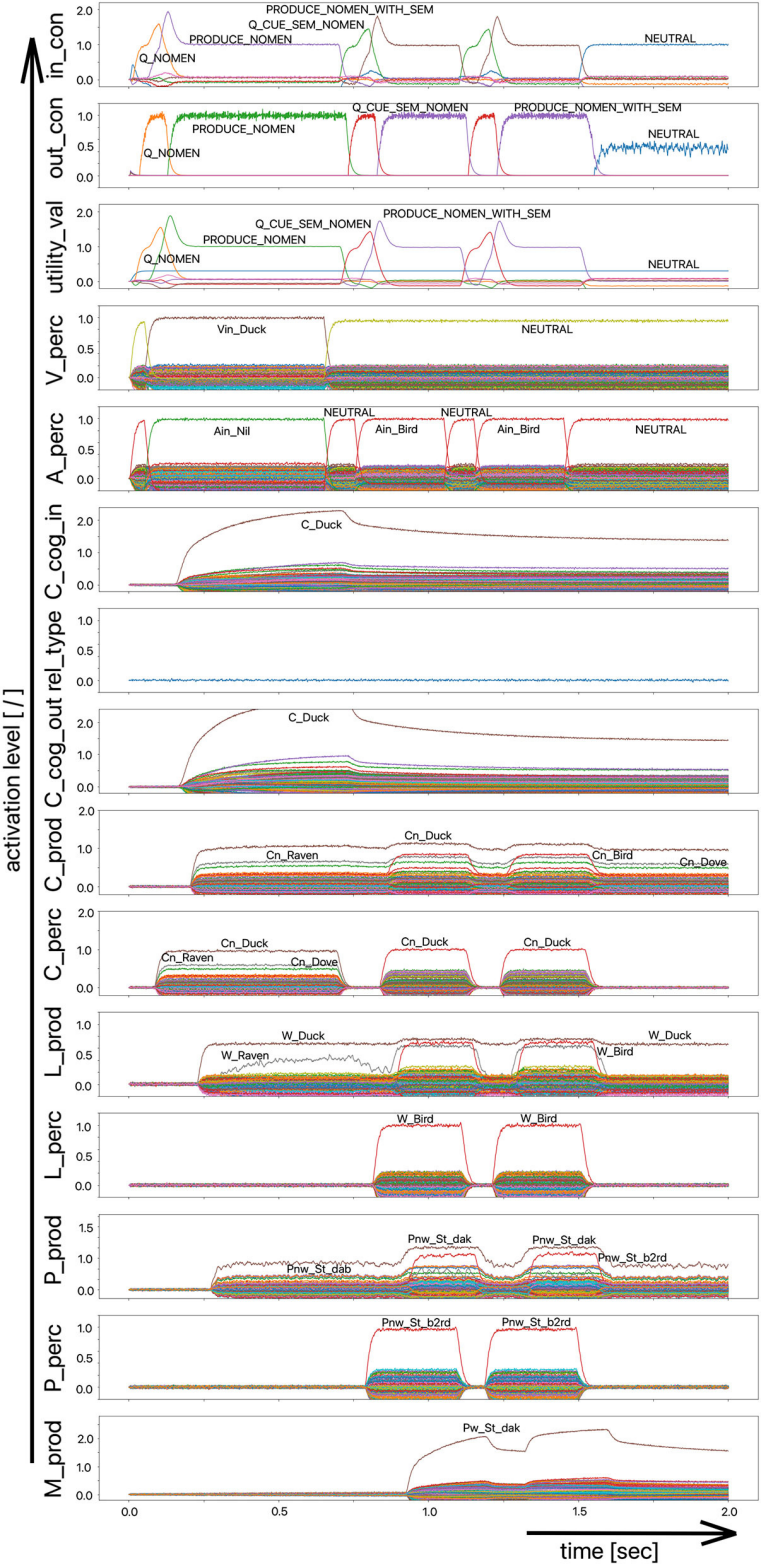
Decoded neural information in the neural state buffers of different model levels on the perception and production side. For a description of the individual buffers, see Figure 2. The process scenario is the picture naming task (target word here: “duck”). After 750 ms, the test supervisor provides an additional semantic cue via the auditory channel [“(the target word belongs to the group of) birds”]. In this example simulation, this leads to the activation of the correct word.

The target words, which are systematically only weakly activated in this experiment within the picture naming time slot, can be compared to the phenomenon that speakers describe colloquially as “the word is on the tip of my tongue.” This situation applies to the initial period of the simulation up to ~750 ms. The simulation experiment is defined such that phonological or semantic help is given in the following time period. Even if the correct word was activated at the lemma level of the production channel due to picture naming in the first 750 ms (see Figure 8), in many cases the activation remains poor in the lower-level state buffers (i.e., below 30% of normal activation on the motor plan level in the first 750 ms for the target word; no activation in case of the examples given in Figures 7, 8) and thus does not lead to activation of the motor plan of the target word. This can be clearly recognized from the simulation examples in Figures 7, 8. But even if in the time window up to 750 ms, before a semantic or phonological cue is introduced, the motor plan of the target word (here: “snake” or “duck”) is not activated, then it becomes fully activated with respect to the later occurring cues.

The adjustment experiment showed that a damping of the neural association between the concept and lemma state buffers by approximately one third is sufficient to meet the precondition of a partly disturbed lexical call. The target word is “on the tip of the speaker's tongue,” but the speaker cannot access it completely during picture naming.

As a result of the occurrence of a phonological cue (/snE/ in Figure 7) or of a semantic cue (“bird” in Figure 8) offered by the test supervisor, the correct target word is activated at the motor plan level in the time period from 750 to 1,500 ms (see: M_prod buffer in Figures 7, 8).

The aim of this simulation experiment is to evaluate the influence of phonological and semantic cues. Indirectly, this simulation experiment is also used to check the S-pointer networks on the concept level and on the level of the phonological forms within the mental lexicon. These networks realize the relations between the S-pointers occurring at these levels and thus determine the similarity relations between concepts or between phonological forms.

Based on the example simulations shown in Figures 7, 8, the activation of the selected word on the lemma level (i.e., an activation of not more than 70% of normal state activation), which was initially too low and leads to no or only very low activation at the motor plan level, is ultimately strengthened now at the motor plan level as a result of the cues provided. The phonological S-pointer network is designed in such a way that, for example, all words that begin with the sound sequence /snE/ have a common similarity relationship (here: the words “snake,” “snail,” and “snack” for Figure 7, see also Table 1). The same applies at the semantic level to all concepts that can be summarized under the same generic term (e.g., under the generic or superordinate term “bird;” here: “duck,” “dove,” and “raven,” for Figure 8, see also Table 1). The construction of an S-pointer network for the phonological form and for the motor plan level inventory used in these simulation experiments (Table 1) is described in detail in Kröger et al. (2016b).

In both cases shown above for phonological and semantic cures, no direct coupling from the perception channel to the production channel was used at the phonological level. In the two examples given above, the processing of the phonological cue is initially carried out via the large processing loop and thus via the concept level. According to Figure 7, this leads to the situation that, based on the phonological cue, all words beginning with /snE/ (“snake,” “snail,” and “snack”) are ultimately activated within the perception channel and then passed on via the concept level toward the production channel. This ultimately strengthens the activation of the target word “snake” in the period after 750 ms on the phonological level as well as on the motor plan level of the production side. Thus, a phonological cue is also successful via the detour of a lexical processing for all phonologically similar words.

**Figure 9:**
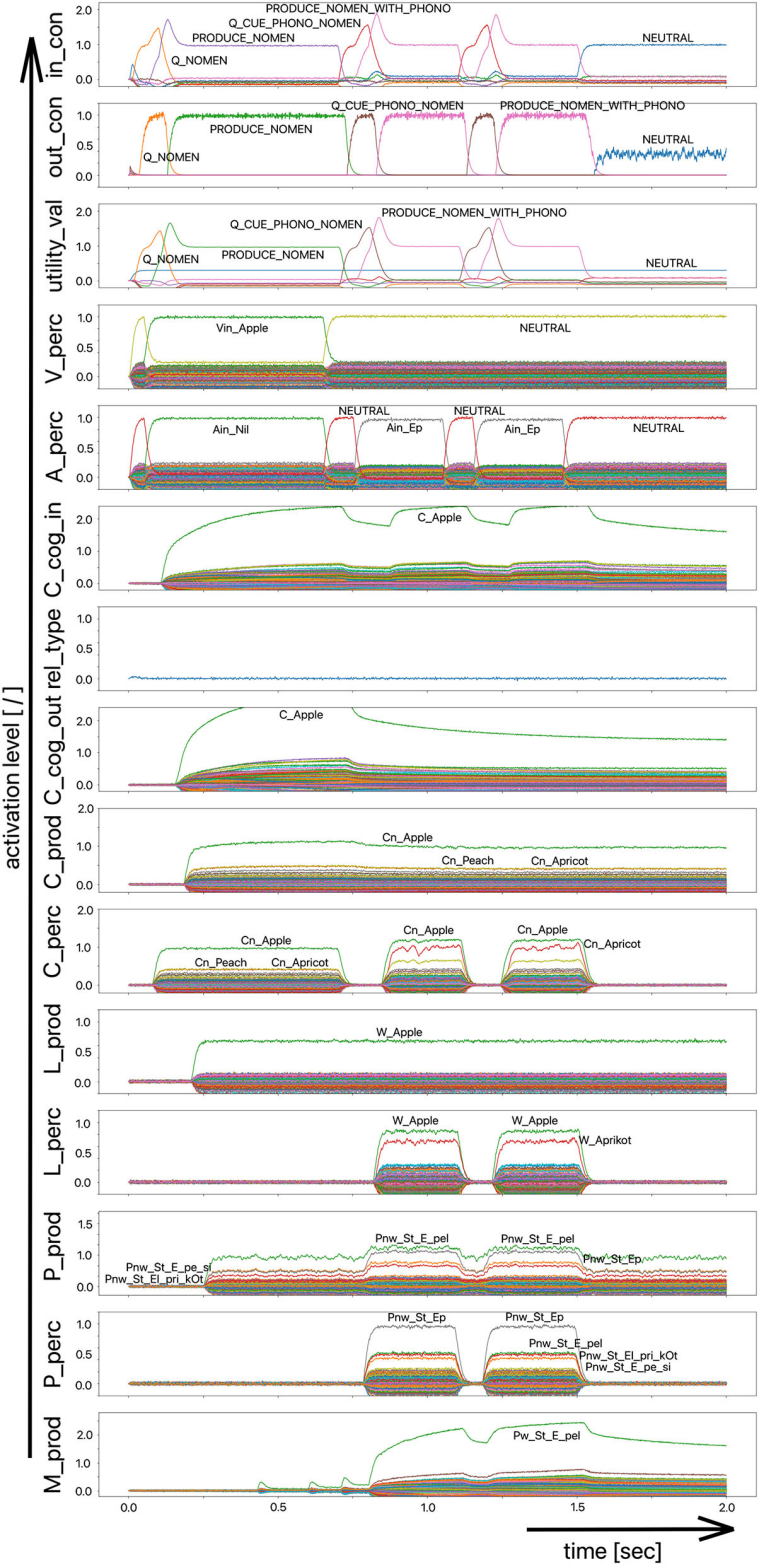
Decoded neural information in the neural state buffers at different model levels on the perception and production side. For the description of the individual state buffers, see Figure 2. The process scenario is a picture naming task (target word “apple”) plus phonological cue (/Ep/) comparable to Figure 7. In contrast to the simulations shown in Figures 7, 8, a model version was chosen here that directly connects the perception to the production side at the phonological level of the model (shortcut P_perc -> P_prod).

However, a simpler processing route for phonological cues is also conceivable, namely the non-lexical route of the direct connection from the perceptual to the production side at the phonological level (P_perc -> P_prod), which has already been discussed in simulation experiment 1. In order to be able to evaluate the influence of this shortcut, a version of the model with that shortcut from the perception to the production side at the phonological level is implemented (simulation example in Figure 9) as well.

It can be seen in Figure 9 from the activation pattern in the phonological state buffer of the production channel that the activation of the word “apple” increases slightly here after 750 ms. While the mean activation before this point in time was ~60–75% of the standard activity, it now increases slightly due to the co-activation of the phonetic form of the word “apple” in the phonological state buffer on the perception side, which helps to increase the activity in the phonological state buffer on the production side to about 90%. While this increase on the phonological level is only very slight, there is a significant increase in the neuronal activation for the target word at the motor plan level (from ~800 ms; Figure 9). It therefore only takes a small impulse, here through the phonological cue, to actually trigger the production of the weakly pre-activated target word.

This third simulation experiment thus consists of three subsets of simulations. (i) Picture naming (18 target words) with phonological cues—model without shortcuts at the phonological level; (ii) Picture naming (18 target words) with phonological cues—model with shortcuts at the phonological level; (iii) Picture naming (18 target words) with semantic cues—model without shortcuts at the phonological level. The simulations for all 18 target words were done three times for each of the three subtests, because the target words are sometimes already fully activated on the motor plan level in the initial time range of the picture naming part of the simulation task. A total of 3 × 18 × 3 = 162 simulations were carried out. The phonological cues are also listed in Table 1 for each target word. The semantic cues are the generic or superordinate terms, which are also listed in Table 1.

## Results

### Experiment 1

**Figure 10:**
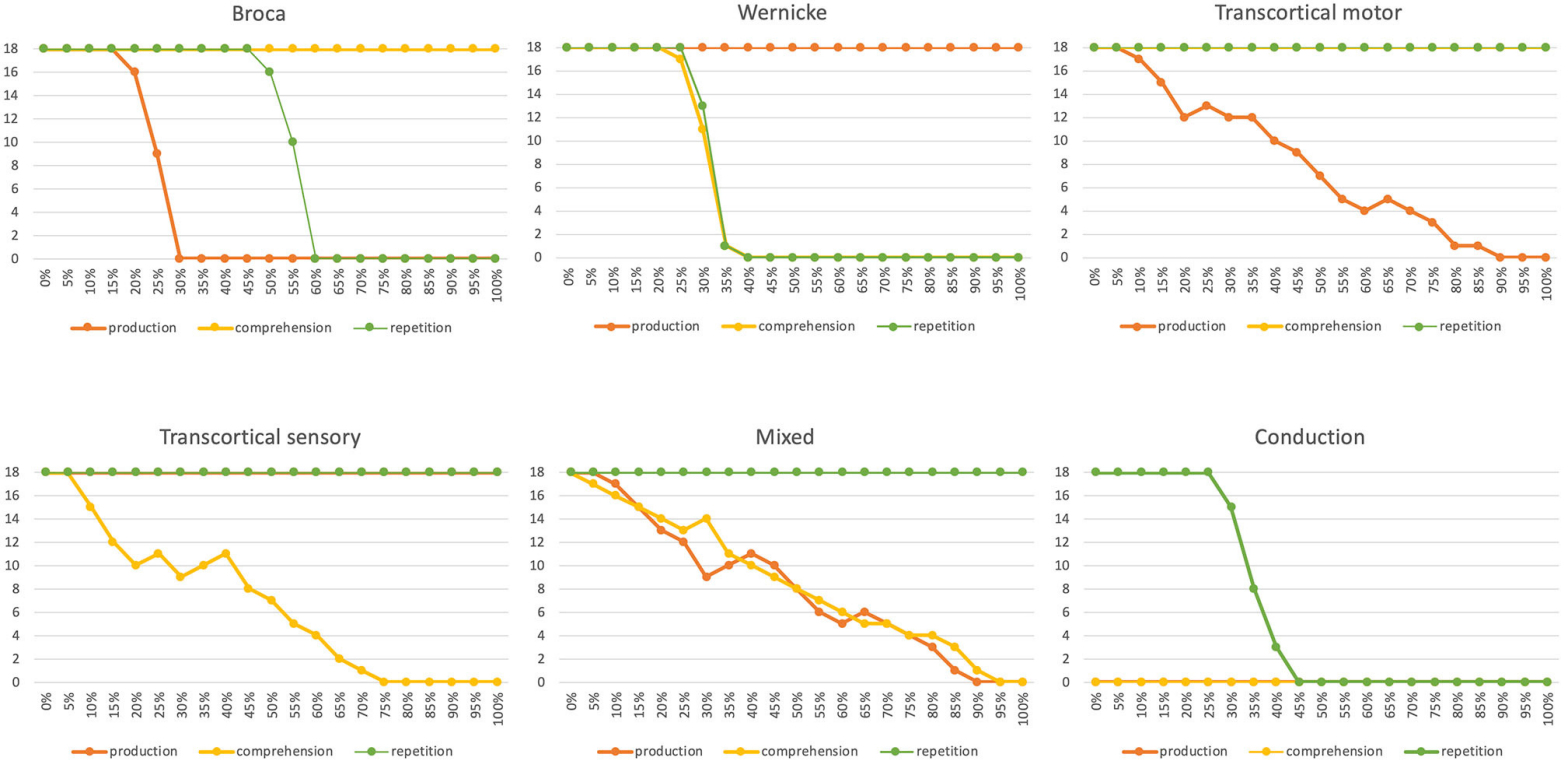
Number of correctly implemented target words (maximum value is 18) as a function of the degree of neural dysfunction for the appropriate state buffer or associative memory perturbed by the speech disorder for three different tasks [picture naming (i.e., production); word comprehension and word repetition] for six different neuronal dysfunctions (Broca, Wernicke, transcortical motor, transcortical sensory, mixed and conduction aphasia). In the case of conduction aphasia, the percentage of neuronal dysfunction corresponds directly to the percentage of weakening the appropriate neuronal connections between P_perc and P_prod, since both memories (P_prod and P_perc) use the same S-pointer activation pattern and therefore no associative memory has to be interposed, but a direct neuronal association is possible. In the case of neuronal dysfunction of a buffer or associative memory, the percentage indicates the percentage of ablated neurons within that buffer or memory.

The simulation results from experiment 1 can be categorized as follows: (i) The picture naming task is successfully completed by the model if the activation of the correct target word in the motor plan buffer is at least 70% of the normal activation (see Figure 2) after the point in simulation time of 300 ms; (ii) The word comprehension task is successfully completed by the model if the activation of the target word in the concept state buffer of the perception channel during the time interval between 150 and 300 ms simulation time is at least 70% of the normal activation, or if the activation of the word characterizing the generic term in the concept buffer of the production channel in the time interval 200–300 ms is at least 70% of normal activation. Since this is a word comprehension task, the full activation of one of the two words at the concept level is sufficient and therefore a verbal realization. In other words, a high activation of the target or word or of the superordinate on all levels within the production channel, is not necessary. (iii) The repetition task is successfully completed by the model if the activation of the target word in the motor plan state buffer after the point in time of 300 ms simulation time is at least 70% of the standard activation. The simulation results were evaluated for each simulation run using the generated plots of S-pointer activations within the appropriate state buffers (see Figures 2–4). The results are summarized in Figure 10. The degree of neuronal dysfunction is given as percentage (0% means no dysfunction and no ablation of neurons, while 100% means all neurons of the corresponding buffer or memory are ablated).

Six different types of neural dysfunctions are simulated: *Broca or Wernicke aphasia*: dysfunction of the phonological state memory on the production or on the perception side; *transcortical motor or transcortical sensory aphasia*: dysfunction of the associative memory between the lemma and concept memory in the production or perception channel; *mixed aphasia*: dysfunction of the neuronal associations between the lemma and word levels in both the perception and production channels; *conduction aphasia*: dysfunction of the neuronal association between the phonological state buffers from the perception to the production side. These dysfunctions are simulated by ablating a certain percentage of the neurons in certain buffers or associative memories in the model. The percentage of ablated neurons varies between 0 and 100%.

It can be seen that in all six cases of different neural dysfunctions, the performance of the simulation drops from 100% (all 18 target words correctly recognized) to 0% (none of the target words was correctly recognized for specific tasks; Figure 10). In the case of Broca aphasia, the production side reacts. At 20–30% ablation of the affected buffer an abrupt decrease in the test performance occurs for the picture naming task, and in the case of 50–60% ablation of the affected buffer an abrupt decrease in test performance occurs for the repetition task. Word recognition remains intact. In the case of Wernicke's aphasia, the perception side changes. Word comprehension and repetition show an abrupt decrease in test performance in the range of 20–40% of the ablation of the affected buffer. The picture naming task is not affected here.

In the case of transcortical motor aphasia, we can find a continuous decrease in test performance over the entire range of ablation from 0 to 100% for the task of picture naming (production). Word comprehension and word repetition remain untouched. In the case of transcortical sensory aphasia, a continuous decrease in test performance can be seen over the entire range of ablation from 0 to 100% for word understanding. Picture naming and repetition remain unaffected. In the case of mixed aphasia we find of a continuous decrease in test performance over the entire range of ablation from 0 to 100% for the tasks of picture naming and word comprehension. Word repetition remains untouched.

In the case of conduction aphasia, the test performance for repetition drops abruptly at 35–45% ablation for the appropriate neural connections between P_perc and P_prod. Word comprehension and picture naming are not affected by this neural dysfunction. However, since we have “uncoupled” the mental lexicon for the implementation of this repetition task, so that the 18 target words in this test series do not result in any neural activation at the lemma or concept levels, the test performance of the remaining two tasks (production and comprehension) is always zero in this simulation experiment.

In summary, it can be seen that in the event of a disruption of neural associations, which are realized by ablating associative memories, the test performance is reduced relatively slowly and continuously during the increase of strength to the neuronal dysfunctions, while the test performance in the case of the dysfunction of a particular state memory occurs abruptly in a small range of ablation values. In other words, while associative memories are relatively robust with regard to neural ablation, this is not the case for state buffers. State buffers implement neural activations of a currently active S-pointer by activating specific sets of neurons within the state buffer. The associated neuronal activation patterns can only be assigned to certain states (certain S-pointers) if only a small percentage of the neurons in this memory are ablated. In the case of association memories, however, the ablation of a certain percentage of neurons only leads to a reduction of the corresponding neuronal activation patterns being passed, i.e., to a signal damping, while the activated S-pointers are passed correctly. The degree of attenuation of the transmitted neuronal signal corresponds approximately to the percentage of ablated neurons within the corresponding associative memory.

### Experiment 2

In the picture naming task with distractor words, a total of two different model variants are tested: (model type 1) To initiate the HALT signal in the control module, both the differences between the type of neural activation of the perception and production side on the phonological level (P_prod/P_perc) as well as at the concept level (C_prod/C_perc) are evaluated by the control module. (model type 2) To initiate the HALT signal in the control module, only the difference between the type of neural activation of the perception and production side is evaluated on the phonological level (P_prod/P_perc). The simulations for each of the 18 target words were implemented with four different types of distractor words and three runs were simulated for each model variant and each target word. The total number of simulations is thus 2 × 3 × 4 × 18 = 432. The simulation results are given in Table 2 for these four different types of distractor words (semantically or phonologically similar to the target word, semantically and phonologically similar to the target word, and semantically and phonologically dissimilar to the target word). To evaluate the simulation results, it is assumed that halting or stopping word production before the start of articulation only takes place if the HALT signal appears strong in amplitude and not later than 40 ms after the starting of the word activation on the motor plan level (see Figures 5, 6).

**Table 2:** Number of triggered stops (HALT signal) in a picture naming task as a function of two different model variants and as a function of the type of distractor word.

**Type of model**	**Type of distractor word**	**Number of stops (test series 1)**	**Number of stops (test series 2)**	**Number of stops (test series 3)**	**Number of stops (sum)**	**Number of simul. Without stop (sum)**
1	Semantic similar	1	2	0	3	51
1	Phonological similar	10	10	9	29	25
1	Sem + phono similar	1	1	0	2	52
1	Dissimilar	17	18	15	50	4
2	Semantic similar	15	15	16	46	8
2	Phonolgical similar	2	2	2	6	48
2	Sem + phono similar	0	1	1	2	52
2	Dissimilar	17	14	13	44	10

*The maximum number of simulations per test series is 18. With a number of stops = 1, no stop is realized in 17 out of 18 simulation cases. The total number of simulations per table row is 54*.

It is striking that dissimilar distractor words are most likely to activate an early HALT signal and thus stop word production. In addition, it can be seen that semantic similarity between target word and distractor word also enables word production to be stopped, while phonetically similar words implement word production in far fewer cases. In addition, it can be seen that the evaluation of the discrepancy of the stimuli between the perception and production side on the phonological level is already very effective for dissimilar as well as for semantic similar distractor words. When including the evaluation of the activation difference within the model buffers at the concept level (model type 2), the simulation results only change with regard to the generation of the HALT signal in case of semantically similarity between target and distractor words.

**Figure 11:**
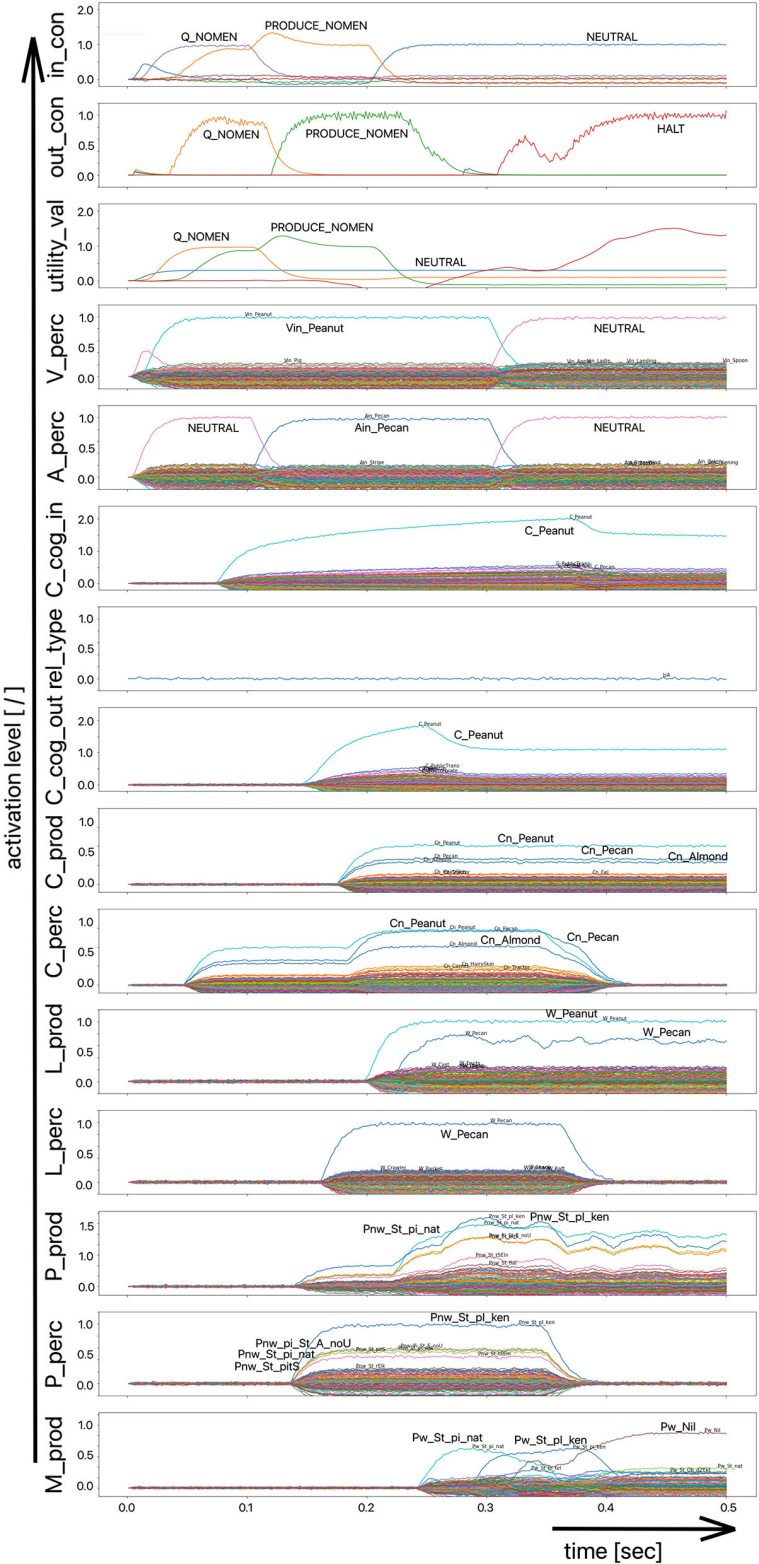
Decoded neural information in the neural state buffers on different model levels on the perception and production side. For a description of the individual state buffers, see Figure 2. The process scenario is a picture naming task (target word here: “peanut”) with an acoustically interspersed word (here: phonologically and semantically similar word: “pecan”). See text for further explanations of this rarely occurring case. In order to give the reader an impression how the association of trajectories and S-pointer names is realized, we here included the labels, automatically generated by the simulation software. (These labels were deleted in Figures 2–9).

In this experiment, speech errors occur in the form of two different words being activated in quick succession (time intervals of not more distance than 50 ms) at the motor plan level (Figure 11). In the case shown in Figure 11, the target word “peanut” is activated on the motor plan level in the time interval 250–320 ms, while the distractor word “pecan” is activated in the time interval 320–380 ms. In total, such a situation occurs in four out of the 432 simulation cases. In two cases, only a semantically similar distractor word is activated. In another two cases, a semantically and phonologically similar distractor word is activated.

### Experiment 3

This simulation experiment comprises three sub-groups of simulations: Picture naming (18 target words) with additional phonological cues (i) without short-circuiting the state memories at the phonological level (lexical route) or (ii) with short-circuiting the state memories at the phonological level and (iii) picture naming (18 target words) with additional semantic cues (model type: no short circuit on the phonological level). The 18 picture naming tasks with additional cues per sub-group were simulated three times for each of the three sub-groups. The results of the total of 3 × 54 = 162 simulations are summarized in Table 3.

**Table 3:** Number of correct target word activation in a picture naming task with phonological or semantic cues before/after occurrence of the cues.

**Number of test series: type of model and type of cue**	**Number of target word activation before/after cue (test-series 1)**	**Number of target word activation before/after cue (test-series 1)**	**Number of target word activation before/after cue (test-series 1)**	**Number of correct target word activation before cue (sum)**	**Number of correct target word activation after cue (sum)**	**Number of activation of incorrect target words (sum)**
1: L-route, phono	8/5	6/6	10/3	24	14	16
2: shortcut, phono	4/6	2/10	8/4	14	20	20
3: L-route, semantic	6/7	4/5	4/4	14	16	24

*The maximum number of simulations per test series is 18. With a number of correct target word activation of 6, correct target word activation was not achieved in 12 out of 18 simulation cases. The total number of simulations per table row is 54. A distinction is made in this table regarding whether a target word is already correctly and completely activated in the motor plan state buffer before cues are given, i.e., within the first 750 ms of the simulation time, or whether the target word was only correctly activated after occurrence of cues. “L route” is model type “lexical route” without short-circuit at the phonological level; “Shortcut”: model type with decoupled mental lexicon, but with short-circuit of the state buffers at the phonological level P_perc -> P_prod*.

In total, wrong words (speech errors) are realized in three of 162 simulation runs. One speech error occurs in the case of phonological cues in the lexical route model. Here the phonological cue leads to a correction of the production toward the correct target word. Two other speech errors occur in the case of simulation runs using semantic cues, whereby in one case the help is then used to implement the cue word itself and in the other case a phonologically similar form is generated first, but then the correct target word is still activated with help of the semantic cue.

**Figure 12:**
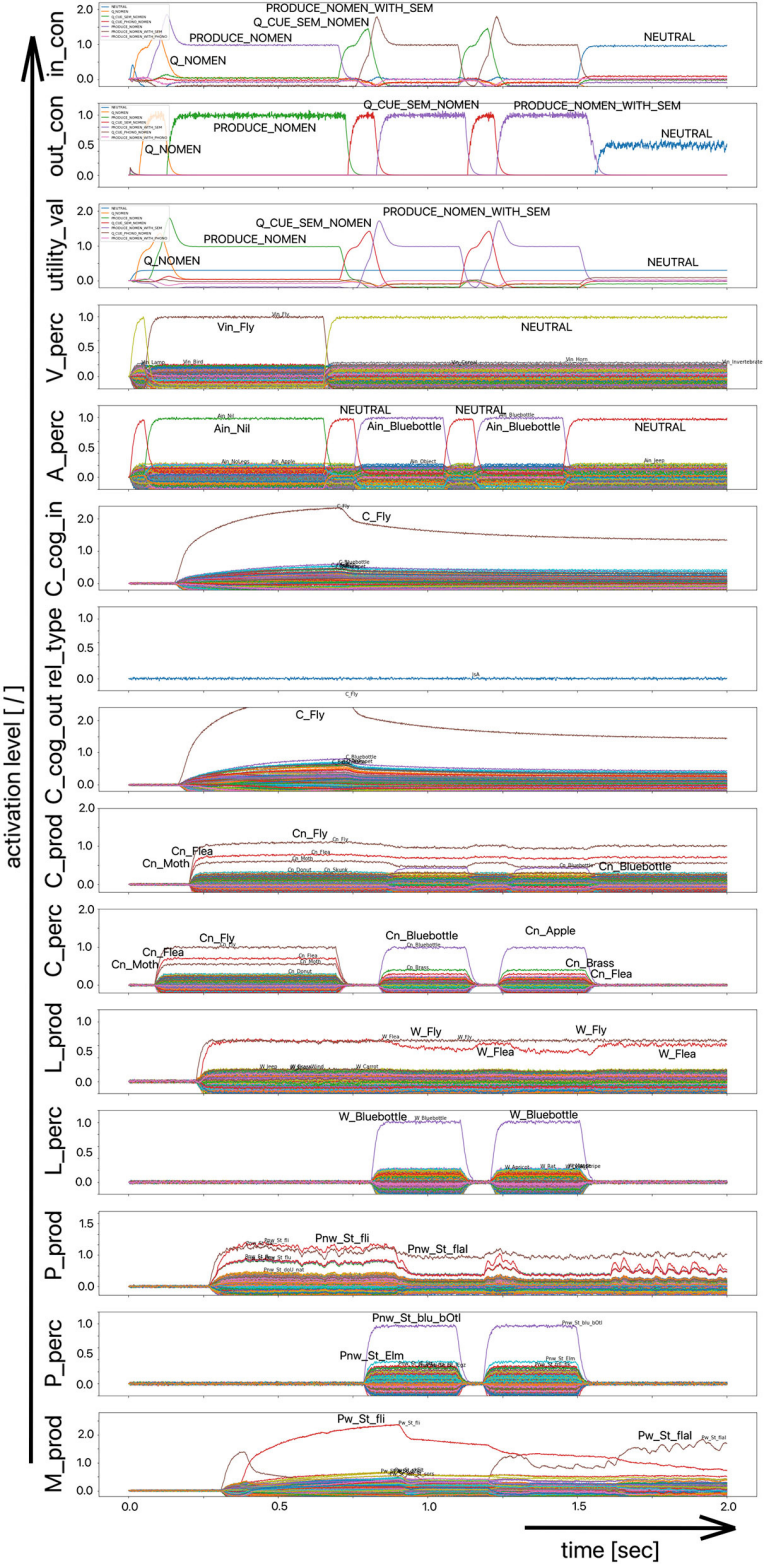
Decoded neural information in the neural state buffers at different model levels on the perception and production side. For the description of the individual state buffers see Figure 2. The process scenario here is a picture naming task (target word here: “fly”) with a later occurring semantic cue, i.e., the superordinate word “bluebottle.” See text for further explanations of this rarely occurring case.

As an example, the simulation of the error in the last simulation run described above is presented in Figure 12. Due to the insufficient activation of the target word at the lemma level within the production channel, the word “flea,” which is phonologically and semantically similar to the target word “fly,” is activated here from ~350 ms. Due to the latter semantic cue, the correct target word is then realized starting with its activation at 1,700 ms in the motor plan buffer.

## Discussion and Outlook

We have implemented a biologically inspired computational model of speech production that is able to simulate both non-disturbed and disturbed speech. The model is structured hierarchically and the neural feedforward activations from the cognitive level to the motor level of speech production are influenced by several feedback mechanisms.

Five main results of this study can be noted:

(i) Neural dysfunctions can be implemented to model certain types of speech disorders. In this paper, six different types of aphasia were modeled. The simulations indicate decreasing speech performance with increasing severity of neural dysfunction. Our simulation results are in line with behavioral data and with the data generated in other simulation studies (cf. Roelofs, 2014). One specific result of experiment 1 is that associative memories are relatively robust with regard to neural ablation, while this is not the case for state buffers. Associative memories are mainly impaired in the transcortical motor area and the transcortical sensory area in the mixed type of aphasia, while state buffers are mainly impaired in Broca's, Wernicke's, and in conduction aphasia. This finding can only be seen in modeling studies and cannot be replicated easily by behavioral experimental studies because the severity of different types of aphasia is mainly clustered (Kang et al., 2010). Here, Broca's aphasia in many cases occur as a moderately severe speech disorder, while all other types of aphasia are clustered as moderate. On the one hand, further studies collecting behavioral data from patients are needed in order to underpin these very specific results stemming from modeling studies in general, but on the other hand it demonstrates that modeling is capable of generating results or knowledge which cannot easily be generated from natural behavioral data, because of the difficulties in finding enough patients covering a wide range in different degrees of severity for different sub-types of aphasia.
(ii) Experimental behavioral results which prove the existence of the inner feedback loop can be simulated in our model as well. For this purpose, the model receives auditory distractor words during a picture naming task. These distractor words are phonologically and/or semantically similar or dissimilar to the current target word. It can be seen in the behavioral experiments (e.g., Slevc and Ferreira, 2006) as well as in our simulation experiments that phonologically dissimilar distractor words in particular disrupt word production. This result can be explained in the context of our speech production model by evaluating the activation differences occurring in the state buffers in the production as well as in the perception channel on the phonological level of our model.
(iii) Speech errors as well as the effectiveness of phonological or semantic cues within a word production task can be simulated in the experimental paradigm of picture naming in our model, if word production is already disturbed in the model by restricting the neural association from the state buffer for concepts to the state buffer for lemmas in the production channel. The resulting effectiveness of the phonological as well as the semantic cues results in our model from the implementation of similarity relations between concepts or between phonological forms in the appertaining S-pointer networks. The effectiveness of semantic as well as phonological cues was proven by natural data, for example in the context of the evaluation of the WWT (Glück, 2011).
(iv) Speech errors arise from the modeling of aphasic neuronal dysfunctions in the context of our simulations only insofar as at higher degrees of dysfunction, words are not activated at different levels of the production hierarchy. This creates Nil productions (a complete stop of word production). These simulations did not show any word productions. The production of wrong words occurred in the other two simulation experiments. To a limited extent, words other than the target words were realized as part of an image naming task due to semantic cues. On the one hand, these “wrong words” were the words of the semantic cues itself or phonologically similar words. Similar types of speech errors also occurred in the case of the simulation experiment on picture naming in the context of auditorily presented distractor words. In this case the erroneous word productions were always the semantically and/or phonologically similar distractor words themselves.
(v) A major innovation of the simulation approach presented here in comparison to other existing approaches (Guenther, 2006; Roelofs, 2014; Kearney and Guenther, 2019) is the use of a biologically more realistic modeling approach (NEF-SPA, Eliasmith, 2013; Stewart and Eliasmith, 2014). Our approach has the advantage over conventional connectionist approaches that a realistic basic model for individual neurons exists (here: the “leaky-integrate-and-fire” approach). This also leads to a realistic modeling of state activations at all levels of the production model. Concepts, lemmas, phonological forms as well as motor plan states are realized through realistic specific neural activation pattern involving all neurons within a neuron buffer, in contrast to simple connectionist approaches wherein every state is realized by an (artificial) physical model node.

However, further work is necessary, especially for the modeling of auditory and somatosensory feedback, which is not implemented in our current model. A successful modeling of these further feedback loops and the successful simulation of behavioral effects they imply for speech production has already been successfully presented in part by Guenther (2006). However, as already described in the theoretical part of this article, our goal is to model the level of the speech movement units in addition to the level of the motor plans in order to differentiate between acoustic feedback to mainly regulate the coordination of speech gestures and somatosensory feedback to mainly control individual speech movements units (cf. Hickok, 2012).

A major question of the research topic of modeling is always: How do concepts from the engineered systems help to understand and inform the biological model? To be more precise in the case of our model of speech production: How do the concepts of the Neural Engineering Framework (Eliasmith and Anderson, 2004; Eliasmith, 2013) extended by the concepts of the Semantic Pointer Architecture (Eliasmith, 2013; Stewart and Eliasmith, 2014) inform our speech production model? This can be answered in three main points:

(i) The NEF-SPA concepts clearly separate knowledge storage and (dynamic) neural processing leading to a clear definition of the mental lexicon in our model. (ii) Only a few model components (buffers, associative memories, connections) are required for neural processing in the NEF-SPA approach, which leads to a clear definition of neural processing in word production. (iii) Neural connections can be realized in a way that leads to a clear definition of hierarchy in our speech production model. (iv) The NEF-SPA approach clearly defines both transformation and binding/unbinding processes, both of which are required for cognitive processing above the linguistic or lexical level. Through this minimalism of concepts offered by the NEF-SPA approach, a clear model of word production can be created, which is able to model normal as well as disrupted word production.

In conclusion, it should be emphasized that our model in particular shows the separation of knowledge stored in long-term memory and the short-term activation and processing of states on the perception side as well as on the production side of speech processing. Long-term knowledge is realized in our model by preset S-pointers on the basis of the internal mathematical model. The setting of these S-pointers happens in reality during speech acquisition. In our approach, which is currently limited to word production, relations between concepts on the semantic as well as between phonological forms of words on the phonological level are realized by establishing S-pointer networks. In addition to this long-term storage of states in the form of S-pointer networks, there is the short-term activation, forwarding and processing of states (S-pointers) from state buffer to state buffer by using mainly association memories for defining the transformation of neural states. The basic neural approach behind the implementation of neural buffers, neural memories, and neural connections is already established here as part of the NEF-SPA and is neurobiologically inspired. Another advantage of our approach is the detailed modeling of control flow based on the neuronal processes taking place in the basal ganglia and in the thalamus (Stewart and Eliasmith, 2014).

Currently, neural learning is not included in our model. Neural connections and S-pointer networks are pre-defined on the basis of statistical principles drawn from the NEF-SPA framework. But this is no disadvantage, because in this paper we described a model of an adult language user and the issue of learning during adulthood is not a topic of consideration here. Thus, speech acquisition should also be included in future developments of our model. While the S-pointer networks are completely pre-defined in their specifications in the current simulation model, a continuation of our simulation model is also planned in such a way that learning can be simulated during defined scenarios of speech acquisition in order to build up the mental lexicon and the mental syllabary during simulations of the child-caretaker interaction.

## Data Availability Statement

The raw data supporting the conclusions of this article will be made available by the authors, without undue reservation.

## Author Contributions

BK did the computer simulations and wrote the paper. CS helped with simulations and with interpretation of simulation results. PB, TB, and TS helped with programming the source code for the simulations and corrected the manuscript. All authors contributed to the article and approved the submitted version.

## Conflict of Interest

The authors declare that the research was conducted in the absence of any commercial or financial relationships that could be construed as a potential conflict of interest.

## Appendix

**Table A1:** Abbreviations, names, and brief description of all state buffers used in our model (see Figures 1, 2 ff).

**Module**	**Buffer**		
	**Abbreviation**	**Name**	**Description**
Control	out_con	Output@control	Output action buffer at control module
	in_con	Input@control	Input signal buffer at control module
Cognitive processing	C_cog_in	ConceptInput@cognitiveProcessing	Concept input buffer at cognitive processing module
	C_cog_out	ConceptOutput@cognitiveProcessiong	Concept output buffer at cognitive processing module
	rel_type	RelationTypeBuffer@cognitiveProcessing	Relation type buffer at cognitive processing module
Production	C_prod	conceptState@production	Concept state buffer at production pathway
	L_prod	LemmaState@production	Lemma state buffer at production pathway
	O_prod	orthographyState@production	Orthography state buffer at production pathway
	P_prod	phonologicalForm@production	Phonological form buffer at production pathway
	M_prod	motorState@production	Motor state buffers at production pathway (gesture score of a syllable or word)
	G_mot	gestureState@motor	Gesture state buffer at motor level of production module (the gesture score of a syllable or word comprises more than one gesture per syllable or word)
Perception	C_perc	conceptState@perception	Concept state buffer at perception pathway
	L_perc	lemmaState@perception	Lemma state buffer at perception pathway
	V_perc	VisualState@perception	Visual state buffer at perception pathway
	P_perc	phonologicalForm@perception	Phonological form buffer at perception pathway
	A_perc	auditoryState@perception	Auditory state buffer at perception pathway
	S_perc	somatosensoryState@perception	Somatosensory state buffer at perception pathway
